# From Agricultural Waste to Sustainable Adsorbent: Characterization of Hazelnut Shell Biochar and Its Performance in Waste Frying Oil Purification

**DOI:** 10.3390/foods15162788

**Published:** 2026-08-08

**Authors:** Ayşenur Özuysal, Şelale Öncü Glaue, Tolga Akcan

**Affiliations:** 1 Department of Environmental Engineering, Faculty of Engineering, Dokuz Eylül University, 35390 İzmir, Türkiye; aysenur.bolukbas@deu.edu.tr; 2 Food Processing Department, Efes Vocational School, Dokuz Eylül University, 35920 İzmir, Türkiye

**Keywords:** biochar, waste frying oil, purification, adsorbent, hazelnut shell, pyrolysis

## Abstract

Agricultural hazelnut shell waste was converted into biochar by slow pyrolysis at 500, 700, and 900 °C and evaluated as a sustainable adsorbent for waste frying oil purification. The biochars were characterized by proximate, thermal, spectroscopic, morphological, and elemental analyses, including Brunauer–Emmett–Teller (BET) surface-area measurement, and benchmarked against activated carbon and Magnesol. Pyrolysis markedly developed the pore structure, increasing the BET surface area from 1.68 m^2^/g in the raw shell to 666.05 m^2^/g at 900 °C, and an exploratory principal component analysis summarized 90.85% of the total variance. Acid-extractable element concentrations were screened against feed-grade biochar and oenological bentonite reference values, without establishing regulatory compliance or food-contact suitability. All three biochars significantly reduced free fatty acidity (27.6–37.8%) and p-anisidine value (23.3–27.5%), with no significant difference relative to activated carbon (Tukey’s test, *p* > 0.05); however, total polar material was not significantly reduced under these mild, short-contact conditions. Gas chromatography showed no significant changes in the major unsaturated fatty acids of the glyceride-bound fraction, and total color differences remained small (ΔE* = 0.71–1.82). Hazelnut-shell biochar therefore shows potential as a waste-derived, circular adsorbent for selected oil-degradation products.

## 1. Introduction

Deep fat frying is one of the most widely used food-cooking methods worldwide. During frying, oil undergoes complex chemical changes under the influence of high temperatures (150–190 °C), moisture, and atmospheric oxygen. The oil serves not only as a heat-transfer medium but also as an ingredient that penetrates the pores of the food and influences its final sensory and nutritional properties [1,2]. Three fundamental degradation reactions occur simultaneously: oxidation, hydrolysis, and polymerization [3]. Oxidation produces peroxides, aldehydes, ketones, and other volatile compounds, while hydrolysis driven by moisture in the food leads to the accumulation of free fatty acids (FFAs) and mono- and diacylglycerols [4]. During prolonged, repeated frying, polymerization produces polymeric triglycerides, and the concentrations of potentially harmful compounds, including trans fatty acids, cyclic fatty acid monomers, sterol oxides, and acrylamide, can increase [5].

Numerous epidemiological and toxicological studies have described adverse effects associated with degraded frying oils. Consumption of repeatedly heated vegetable oils has been associated with increased blood pressure and total cholesterol, vascular inflammation, atherosclerosis, and a greater risk of cardiovascular disease [6,7]. In vivo studies have also shown that lipid peroxidation products can induce oxidative stress and damage liver and gastrointestinal tissues [8]. Ganesan et al. [6] further discussed the occurrence and health relevance of polycyclic aromatic hydrocarbons in repeatedly used frying oils. Acrylamide, formed through reactions involving carbonyl compounds and amino acids, particularly asparagine, at high temperature [9], has been classified by the International Agency for Research on Cancer as probably carcinogenic to humans (Group 2A) [10]. Regulatory criteria for frying-oil disposal vary among jurisdictions; reported maximum total polar material (TPM) values are generally 24–27%, including 24% in Germany and 25% in Spain, France, and Belgium [11,12]. Japanese guidance has also used an acid value threshold of approximately 2.5–3.0, while Belgium has applied a viscosity limit of 37 mPa·s at 50 °C [11,12].

Various adsorbent technologies have been developed to improve frying-oil quality and extend service life. Synthetic magnesium silicate (Magnesol XL), activated carbon, bleaching earth, diatomaceous earth, and other silicate-based adsorbents are commonly used for this purpose. Yates and Caldwell [13] compared several adsorbents for removing polar degradation compounds and reported an adsorption capacity of approximately 200 mg/g for magnesium silicate, compared with 2 mg/g for diatomaceous earth. The performance of magnesium silicate has been associated with basic active sites that facilitate adsorption of acidic polar compounds [13,14]. Lin et al. [15] found that an HB 600–Magnesol combination reduced FFA by 90.8–93.7%, and Bhattacharya et al. [16] reported that binary and quaternary adsorbent mixtures outperformed individual adsorbents in used palm oil. Maskan and Bagci [17] identified a combination of pekmez earth, bentonite, and magnesium silicate as the best treatment among those tested, while Turan and Yalcuk [18] reported that silica gel achieved a 100% improvement in TPM in a column process. Adsorbent-based refining has also been applied to other vegetable oils: silicon dioxide was used to degum crude rapeseed oil while retaining up to 96% of its phenolic content and preserving its fatty-acid profile [19], and Al_2_O_3_ nano-adsorbents combined with electrostatic-field treatment reduced the acid value of crude canola oil by 62.4% [20]. However, commercial adsorbents add material costs, and spent media require disposal or regeneration; waste-derived biochars may offer a more circular alternative [21].

In this context, biochar produced from agricultural waste through pyrolysis has emerged as a promising alternative adsorbent because of its tunable pore structure and surface chemistry [21]. Studies on biochar-based materials for edible-oil purification have recently increased. Turan et al. [22] demonstrated that activated carbons produced from horse chestnut, chestnut, acorn, pistachio, and apricot kernel shells could improve TPM by 100% and p-anisidine value by 92% in used frying oil. Chathiran et al. [23] found that corncob biochar reduced acrylamide and improved oxidation parameters in repeatedly used palm oil. Abdelhameed et al. [24] reported that a ZIF-8@biochar composite removed FFA by 80.6%, peroxide value by 70.6%, and acrylamide by 99.9% from frying oil.

Türkiye is the world’s leading hazelnut producer, accounting for approximately 62–75% of global output, with annual production typically ranging from 600,000 to 800,000 tonnes [25]. During industrial processing, shells constitute approximately 42–55% of the fruit mass, depending on the variety, and consequently generate a substantial lignocellulosic residue [26]. Hazelnut shells contain approximately 25–30% hemicellulose, 26–32% cellulose, and 40–43% lignin [27]. Their high lignin content favors the formation of a stable carbonaceous residue during pyrolysis, while their low ash content may be advantageous where excessive mineral loading is undesirable [28,29]. Converting this residue into value-added products is therefore relevant to circular-economy management [30].

Studies on hazelnut shell biochar have shown that as the pyrolysis temperature increases, biochar yield decreases, while fixed carbon content and porosity increase [29]. Biochar has shown BET surface areas of 124.35 m^2^/g at 500 °C pyrolysis and 197.32 m^2^/g at 800 °C [28,29]. With chemical activation, these values can be increased substantially; Guo et al. [31] achieved an exceptionally high surface area of 2979.59 m^2^/g through ZnCl_2_ impregnation and KOH activation. Moreover, in another study, the BET surface area increased to 1413.02 m^2^/g via activation with ZnCl_2_ and FeCl_3_ [32]. Although the effectiveness of hazelnut shell biochar has been demonstrated in various adsorption applications, including heavy metal removal [33], dye removal [28], and pharmaceutical pollutant removal [32], to the best of our knowledge, this study presents the first evaluation of hazelnut shell biochar as an adsorbent for waste frying oil purification, benchmarked directly against two commercial adsorbents (activated carbon and Magnesol).

Based on this critical knowledge gap, the present study aims to conduct a comprehensive characterization (BET, SEM-EDS, FT-IR, ICP-OES, TGA-DTA, and DSC) of biochar produced from hazelnut shells at different pyrolysis temperatures and to evaluate its effectiveness as an adsorbent for the purification of used frying oil. This work contributes to the valorization of hazelnut shells, one of Türkiye’s significant agricultural waste streams, as a sustainable adsorbent for the food industry.

## 2. Materials and Methods

### 2.1. Raw Material

Hazelnut shells were used as the biomass feedstock. The locally sourced material was cleaned to remove visible foreign matter and dried in an oven at 105 ± 5 °C for 24 h. The raw shells used under the reference pyrolysis condition were 15–20 mm in size.

### 2.2. Proximate Analysis

The basic physicochemical properties of the raw hazelnut shells were determined by proximate analysis. Moisture and organic-matter contents were determined in accordance with TS ISO 11465 [34] and ASTM D2974-13 [35], respectively. Volatile matter and ash were measured according to ASTM E872-82 [36] and ASTM E1755-01 [37], respectively. Fixed carbon was calculated by difference on a dry-weight basis [38]. All proximate analyses were conducted in triplicate.

### 2.3. Thermal Analyses (TGA-DTA and DSC)

The thermal behavior of raw hazelnut shells was investigated by thermogravimetric analysis (TGA) and differential thermal analysis (DTA) using a PerkinElmer STA 6000 (PerkinElmer, Waltham, MA, USA). Approximately 10 mg of sample was placed in a platinum crucible and heated from room temperature to 950 °C at 10 °C/min under nitrogen flowing at 20 mL/min. These analyses were used to identify temperature-dependent mass losses and thermal-decomposition stages.

Thermal transitions and heat-flow changes in the biomass were additionally evaluated by differential scanning calorimetry using a TA Instruments DSC 250 (TA Instruments, New Castle, DE, USA). Approximately 10 mg of HS-Raw was placed in a Tzero hermetic aluminum pan (TA Instruments, New Castle, DE, USA) and heated from room temperature to 600 °C at 10 °C min^−1^ under a nitrogen purge flow of 50 mL min^−1^. The instrument was calibrated using an indium standard before analysis. The DSC data were used to evaluate endothermic and exothermic events occurring during heating.

### 2.4. Biochar Production

The biochar samples were produced by slow pyrolysis under nitrogen flow (1.5 ± 0.3 L/min). The final pyrolysis temperatures were 500, 700, and 900 °C. Unless otherwise specified, the heating rate was 10 °C/min and the residence time at the final temperature was 120 min.

To examine selected operating variables, additional experiments were conducted at 700 °C by changing one condition at a time relative to the reference condition: heating rate (5 °C/min), residence time (60 min), or initial particle size (5–10 mm). The samples were designated HS-500, HS-700, HS-900, HS-700 (5–10 mm), HS-700 (5 °C/min), and HS-700 (1 h).

After pyrolysis, the biochar samples were cooled to room temperature under an inert atmosphere and stored in airtight containers before analysis. Each biochar sample (including the residence-time, heating-rate, and particle-size variants at 700 °C) was produced via a single, independently conducted pyrolysis run under the specified conditions.

### 2.5. Surface Area and Pore Structure Analysis (BET)

The specific surface area and pore characteristics of the samples were determined by nitrogen (N_2_) adsorption at 77 K using a Micromeritics 3Flex surface-area and porosity analyzer (Micromeritics Instrument Corporation, Norcross, GA, USA; İYTE Materials Research Center, İzmir, Türkiye). Before analysis, the samples were degassed at 250 °C for 4 h. BET surface area was calculated over the relative-pressure range P/P_0_ = 0.05–0.30. Total pore volume was obtained from the single-point adsorption value near saturation, and average pore diameter was calculated as D = 4V/A, where V is total pore volume and A is BET surface area.

### 2.6. Fourier Transform Infrared Spectroscopy (FT-IR)

Functional-group analyses of the raw biomass and biochar samples were performed using Fourier transform infrared spectroscopy (FT-IR; Shimadzu IRAffinity, Shimadzu Corporation, Kyoto, Japan). Spectra were recorded over the wavenumber range 4000–650 cm^−1^ at a resolution of 4 cm^−1^ using 25 scans. The spectra were used to evaluate changes in chemical structure as a function of pyrolysis temperature and process conditions.

### 2.7. Morphological and Elemental Analyses (SEM-EDS and ICP-OES)

Surface morphology was examined using a GeminiSEM 560 scanning electron microscope (Carl Zeiss Microscopy Deutschland GmbH, Oberkochen, Germany). Images acquired at different magnifications were used to evaluate pore formation and structural changes qualitatively.

Localized elemental composition was examined by energy-dispersive X-ray spectroscopy (EDS) integrated with the SEM. EDS measurements were performed at an accelerating voltage of 10 kV and a working distance of approximately 9–10 mm, without applying a conductive coating. One representative spot was selected for each sample; consequently, the reported elemental composition should be regarded as a localized, semi-quantitative measurement rather than a bulk-averaged value. The relative percentages of carbon, oxygen, potassium, and calcium were determined for descriptive comparison among samples.

For descriptive comparison of the localized elemental composition, EDS-derived weight percentages (wt.%) were converted to atomic percentages (at.%) using the standard atomic weights of the detected elements (C, 12.011; O, 15.999; K, 39.098; and Ca, 40.078 g mol^−1^). The *O/C* atomic ratio was calculated using Equation (1) [39].

(1) $$O/C\;ratio=\frac{\mathrm{atomic}\;O}{\mathrm{atomic}\;C}$$

Acid-extractable elemental concentrations were determined by inductively coupled plasma optical emission spectrometry (ICP-OES) after microwave-assisted acid extraction using a protocol adapted from USEPA Method 3051A [40]. Approximately 0.5 g of each ground sample was weighed into a PTFE vessel, and 9 mL of HNO_3_ (65%) and 3 mL of HCl (37%) were added (trace-metal grade, Sigma-Aldrich, St. Louis, MO, USA). Extraction was carried out in a closed-vessel microwave system (ETHOS Easy, Milestone SpA, Sorisole, Italy) at 200 °C for 15 min following a 15 min ramp at a maximum power of 1500 W. After cooling, the extracts were diluted to 50 mL with ultrapure water (Milli-Q®, Merck Millipore, Burlington, MA, USA) and filtered through 0.45 µm PTFE syringe filters. Procedural blanks were processed with each batch. Elemental concentrations were measured using a PerkinElmer Avio 200 ICP-OES (Waltham, MA, USA) in axial view with the manufacturer’s prevalidated multi-element method in Syngistix™ for ICP software, version 3.0 (PerkinElmer, Waltham, MA, USA). One extraction and one ICP-OES determination were performed for each material, and the results were reported on a dry-weight basis (mg/kg).

USEPA Method 3051A is an acid-extraction procedure rather than a total-decomposition method [40], and carbonized matrices may resist complete wet digestion [41]. Accordingly, the values reported here represent acid-extractable concentrations rather than total elemental contents and are used only for a preliminary compositional comparison with the cited reference values.

### 2.8. Statistical Analysis

To quantify the strength and direction of the linear relationships between the pyrolysis process variables (temperature, heating rate, and residence time) and biochar properties, a Pearson correlation analysis was conducted in OriginPro 2018 (OriginLab Corporation, Northampton, MA, USA). Two-tailed significance tests were performed at a confidence level of 95% (*p* < 0.05). The obtained Pearson correlation coefficients (r) were visualized as a correlation matrix heatmap to systematically examine the relationships among the parameters before the multivariate modeling. Because the pyrolysis process variables (temperature, particle size, residence time, and heating rate) were varied one at a time around a central condition (700 °C, 120 min, 10 °C/min, 15–20 mm) rather than in a full factorial design, correlations among these variables partly reflect this experimental structure rather than an independent physical relationship. The raw hazelnut shell (HS-Raw) was assigned to a nominal temperature of 25 °C (ambient) and a residence time and heating rate of 0, consistent with the absence of a pyrolysis step. Given that 78 pairwise comparisons were performed relative to the sample size (n = 7), a Bonferroni-corrected significance threshold (α = 0.05/78 ≈ 0.00064) was additionally applied for confirmatory interpretation.

Principal component analysis (PCA) was performed as an exploratory data-analysis tool using OriginPro 2018. The analysis was based on the correlation matrix of the operational parameters and biochar characteristics. Input variables were temperature, residence time, heating rate, biochar yield, BET surface area, and atomic carbon content. Two principal components (PC1 and PC2) were extracted to construct a two-dimensional biplot. A loading magnitude of 0.40 was used as a practical threshold for identifying salient variable–component relationships.

### 2.9. Waste Frying Oil Preparation

Waste frying oil (WFO) was generated under controlled laboratory conditions to obtain a consistently degraded substrate. Commercial sunflower oil was used as the frying medium. Potato strips (6 × 7.8 × 49.5 mm^3^) were fried in an industrial 5 L fryer (Öztiryakiler, İzmir, Türkiye) in 100 g portions for 6 min each, and a total of 50 consecutive frying cycles were performed with the same oil batch without replenishment, yielding a heavily degraded waste frying oil. The oil was then cooled, filtered through filter paper to remove food debris, and stored at 4 °C in amber glass bottles until analysis.

### 2.10. Adsorbents and Adsorption Treatment

The three hazelnut-shell biochars produced at 500, 700, and 900 °C (HS-500, HS-700, and HS-900) were evaluated as adsorbents for the purification of waste frying oil. Two commercial adsorbents were included as benchmarks: a food- and laboratory-grade powdered activated carbon (AC), obtained from a local chemical supplier, and a magnesium silicate-based adsorbent (Magnesol^®^, The Dallas Group of America, Whitehouse, NJ, USA), the latter of which is widely used for industrial frying-oil filtration. To ensure a fair comparison, all biochars and commercial adsorbents were ground and sieved to a uniform particle size of <150 µm (US 100 mesh), consistent with the powder fraction typically used in frying-oil purification [16,42].

The adsorption treatment was carried out under conditions selected to reflect the warm, short-contact active filtration commonly applied in practice. For each treatment, 2 g of adsorbent (4%, *w*/*w*) was added to 50 g of waste frying oil, and the mixture was stirred at 60 °C for 15 min. The solid phase was subsequently removed by centrifugation (4200 rpm, 15 min, room temperature) followed by filtration. A control group (WFO) consisting of the waste frying oil subjected to the same temperature and time conditions without adsorbent addition was processed in parallel. Six groups were thus obtained: WFO (control), HS-500, HS-700, HS-900, AC, and Magnesol. Three independent adsorption treatments were performed for each group using separately prepared aliquots of the waste frying oil, and oil quality parameters were determined for each independently treated sample.

### 2.11. Oil Quality Analyses

Treated and untreated oil samples were characterized using the following quality parameters. Free fatty acidity (FFA; AOCS Ca 5a-40, titrimetric), peroxide value (PV; AOCS Cd 8-53, iodometric titration), specific extinction coefficients (K_232_ and K_268_; AOCS Ch 5-91, measured on a Shimadzu UV-Vis spectrophotometer (Shimadzu Corporation, Kyoto, Japan) using isooctane as the solvent and a 1 cm quartz cuvette, with sample dilution adjusted to keep the absorbance within 0.1–0.8), and p-anisidine value (AnV; AOCS Cd 18-90, colorimetric) were determined according to the corresponding AOCS Official Methods [43]. Total polar material (TPM) was measured with a Testo 265 deep-frying oil tester (Testo SE & Co. KGaA, Titisee-Neustadt, Germany) based on the dielectric-constant principle. Oil color was recorded with a HunterLab colorimeter (Hunter Associates Laboratory, Inc., Reston, VA, USA) in the CIE L*, a*, b* color space, and the total color difference (ΔE*) of each treated sample relative to the untreated control was calculated. All analyses were performed in triplicate.

Because informal single-point measurements of the untreated waste frying oil occasionally exceeded the upper measuring limit of the Testo 265 sensor (0.5–40% TPM), its TPM was determined via a serial dilution series with fresh sunflower oil at defined volumetric ratios (0, 20, 40, 60, 80, and 100% waste oil, *v*/*v*), with each mixture measured in triplicate (n = 18 total measurements). A linear regression of TPM against waste-oil fraction was highly significant (TPM = 7.92 + 0.2805 × fraction (%); R^2^ = 0.996, *p* < 0.001), confirming a linear sensor response across the full compositional range. The undiluted (100%) waste oil fell within the validated measuring range and yielded a TPM of 36.00 ± 1.00% (n = 3), closely corroborated by the regression-based estimate (35.97%; 95% CI: 35.43–36.51%); this value was adopted as the TPM of the untreated control.

Fatty acid composition was determined by gas chromatography after conversion of the oil samples to fatty acid methyl esters (FAME). For methylation, 1 g of sample was weighed and mixed with 2 mL of heptane, then 0.2 mL of methanolic potassium hydroxide solution was added; the resulting supernatant containing the methyl esters was transferred to vials for gas chromatography injection. Analyses were performed on a Shimadzu gas chromatograph (Shimadzu Corporation, Kyoto, Japan) equipped with a flame ionization detector (FID), an AOC-20i autosampler, and a Thermo Scientific TR-CN100 capillary column (Thermo Fisher Scientific, Waltham, MA, USA; 100% cyanopropyl polysiloxane; 100 m × 0.25 mm × 0.20 µm). The injector was operated in split mode (1:100) at 250 °C using hydrogen as the carrier gas (1.20 mL/min, 29.1 cm/s linear velocity), and the FID was maintained at 260 °C (H_2_ 40 mL/min, air 400 mL/min). The injection volume was 1.0 µL. The oven program was 165 °C held for 15 min, then ramped at 5 °C/min to 200 °C and held for 39 min (total run time 61 min). Individual FAMEs were identified against an external FAME standard (Supelco 37 Component FAME Mix, CRM47885, Sigma-Aldrich/Merck, Darmstadt, Germany) and reported as relative area percentages. Fatty acid analyses were performed in triplicate. It should be noted that this methylation procedure converts glyceride-bound fatty acids efficiently but does not methylate free fatty acids; consequently, in the high-FFA waste frying oil, the FFA fraction is not reflected in the reported profile, and the relative composition is evaluated on a glyceride-bound basis.

For the oil-purification experiments, all quality parameters (FFA, PV, K_232_, K_268_, AnV, TPM, color, and fatty-acid composition) were expressed as mean ± standard deviation for three independently treated samples per group. Differences among the six treatment groups were evaluated by one-way analysis of variance (ANOVA), followed, where appropriate, by Tukey’s honestly significant difference (HSD) test (*p* < 0.05). For the complementary multivariate assessment, Pearson correlations were calculated from the 18 replicate-level observations for FFA, PV, K_232_, K_268_, AnV, and TPM; a Bonferroni threshold of α = 0.05/15 ≈ 0.0033 was applied to the 15 pairwise comparisons. An exploratory PCA was performed separately on the standardized group means (n = 6) of the same variables. Statistical analyses of the oil-quality data were performed using IBM SPSS Statistics version 30 (IBM Corp., Armonk, NY, USA).

## 3. Results and Discussion

### 3.1. Raw Material Properties and Proximate Analysis

The physicochemical properties of raw hazelnut shells (moisture, organic matter, volatile matter, ash, and fixed carbon contents) are presented in Table 1. The relatively low water content (7.80%) reduces the additional energy required for water removal during pyrolysis [28,44]. The high organic matter content (98.90%) confirms the predominantly organic, low-mineral nature of the raw material and is consistent with its low ash content (1.10%). The high volatile matter content (80.06%) further indicates a predominantly lignocellulosic structure, and the release of volatile compounds during pyrolysis can contribute to pore development [28,29].

The low ash content (1.10%) indicates that the inorganic fraction of the raw material is small, which can be advantageous for pore development and adsorption performance [29]. In frying-oil purification, minimizing the transfer of mineral impurities from an adsorbent into the oil phase is also desirable [45].

The fixed-carbon content of the raw shell was 18.84%. Together with the high volatile-matter and low ash contents, this result indicates that the feedstock contains a substantial combustible organic fraction capable of forming a carbonaceous solid during pyrolysis [28,29]. The proximate-analysis results therefore support the use of hazelnut shell as a biochar precursor, while adsorption performance is evaluated directly in the later oil-treatment experiments.

### 3.2. Thermal Behavior (TGA–DTA and DSC Analyses)

**Figure 1:**
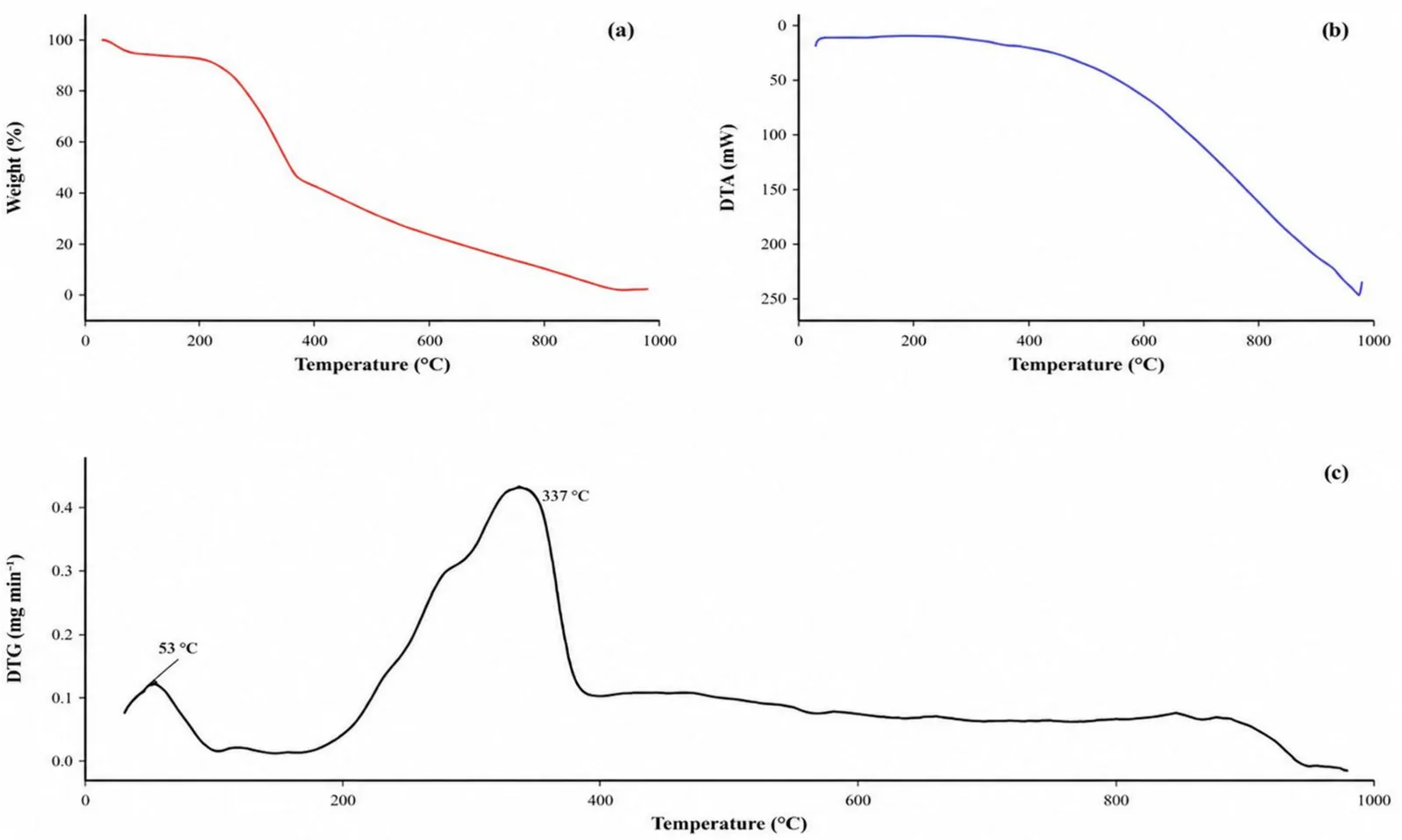
Thermal behavior of raw hazelnut shell (HS-Raw) under nitrogen (20 mL/min) at a heating rate of 10 °C/min: (**a**) thermogravimetric analysis (TGA), (**b**) differential thermal analysis (DTA), and (**c**) derivative thermogravimetry (DTG). The red, blue, and black curves represent TGA, DTA, and DTG, respectively. The DTG curve was calculated from the recorded mass-loss data using the nominal initial sample mass of 10 mg and a locally fitted derivative. The principal DTG maxima occurred at approximately 53 and 337 °C.

The TGA curve of HS-Raw (Figure 1a) showed an initial mass-loss stage below 150 °C, accompanied by a low-temperature DTG maximum at approximately 53 °C (Figure 1c), which was attributed primarily to the removal of moisture and light volatile compounds. The principal devolatilization stage occurred between approximately 200 and 400 °C, with a maximum degradation rate at approximately 337 °C. This broad stage is consistent with overlapping hemicellulose and cellulose decomposition together with the initial degradation of lignin [46,47]. Above 400 °C, the DTG signal remained relatively low and broad without a distinct additional maximum, consistent with slower lignin degradation and progressive rearrangement of the carbonaceous residue [46,47].

Figure 1b presents the DTA heat-flow signal of HS-Raw. A relatively small thermal event below 150 °C coincided with the initial mass loss and was attributed mainly to moisture removal. A more pronounced heat-flow change between approximately 250 and 400 °C corresponded to the principal TGA mass-loss region and the DTG maximum, supporting the overlapping decomposition of the major lignocellulosic components. Above 400 °C, the heat-flow signal changed gradually without a distinct additional peak, consistent with broad, slower transformations of lignin-derived and carbonaceous components [46,47].

**Figure 2:**
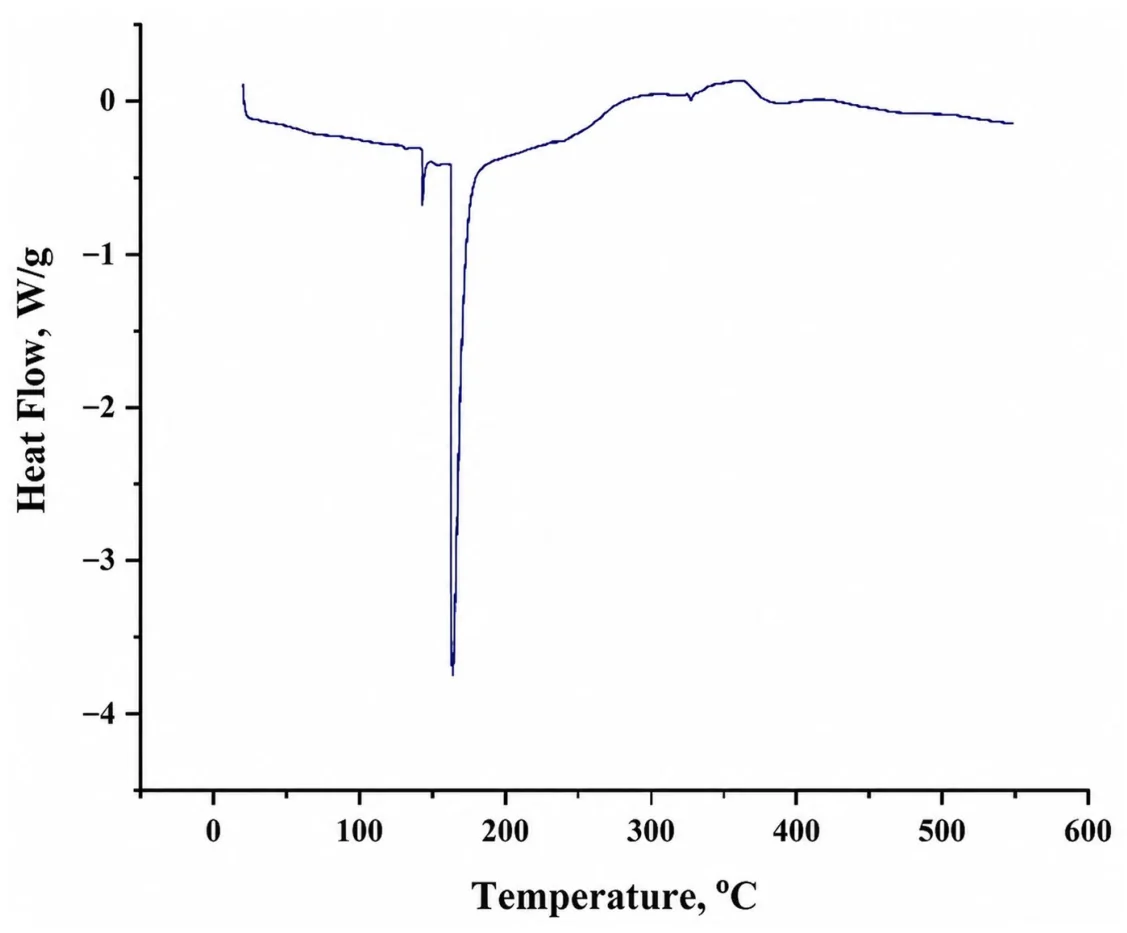
Differential scanning calorimetry (DSC) curve of raw hazelnut shell (HS-Raw), recorded from room temperature to 600 °C at 10 °C min^−1^ under nitrogen (50 mL min^−1^) using a Tzero hermetic aluminum pan.

The DSC curve of HS-Raw is presented in Figure 2. A broad low-temperature endothermic event around 100 °C was attributed to moisture-related processes under the sealed-pan conditions. A sharp endothermic feature was observed near 160 °C. Endothermic events in this range have been reported for plant-derived materials and are associated with overlapping thermal transitions of low-molecular-weight carbohydrates and extractive compounds [48]. In particular, melting-related endotherms of monosaccharides have been reported at approximately 159–170 °C, while hazelnut-shell extracts have exhibited endothermic transitions between 130 and 250 °C associated mainly with polyphenolic constituents [49]. The feature near 160 °C was therefore tentatively attributed to overlapping transitions of native low-molecular-weight extractives rather than to decomposition of a specific lignocellulosic component. Post-run inspection confirmed that the hermetic pan remained intact, with no visible leakage or deformation. The broader heat-flow changes between approximately 250 and 400 °C were interpreted qualitatively, together with the TGA and DTG results, as part of the principal lignocellulosic decomposition region [46,47]. No distinct thermal peak was observed above 400 °C.

The residual mass recorded at the end of the analytical TGA run was lower than both the fixed-carbon value calculated from the proximate analysis and the biochar yield obtained in the tube furnace (Table 2). These quantities are not directly equivalent: fixed carbon is a calculated difference, whereas TGA residue and reactor biochar yield are operationally defined measurements. Differences in sample mass, bed configuration, purge-gas contact, and vapor–solid secondary reactions may have contributed to the observed discrepancy. Therefore, the TGA and DTG data are interpreted here primarily in terms of decomposition stages and characteristic temperatures rather than as quantitative predictors of tube-furnace biochar yield.

### 3.3. Biochar Yields

Biochar yields at different temperatures and operating conditions are shown in Table 2. Across the temperature series, yield decreased monotonically from 34.39% at 500 °C to 20.97% at 900 °C, consistent with greater devolatilization at higher final temperatures [29,50]. Because each pyrolysis condition was represented by one independently conducted run, the trend is interpreted descriptively rather than as a replicated statistical effect.

The secondary process variables (particle size, residence time, and heating rate) were associated with smaller changes in yield than the final pyrolysis temperature, while also affecting the BET and SEM characteristics (Section 3.4 and Section 3.6). The sample produced at the lower heating rate (5 °C/min) had the second-highest biochar yield. Slower heating can prolong vapor–solid contact and favor secondary char formation or recondensation, which may have contributed to this result [51].

### 3.4. Surface Area and Pore Structure Analysis (BET)

BET results for HS-Raw, HS-500, HS-700, HS-900, HS-700 (5–10 mm), HS-700 (5 °C/min), and HS-700 (1 h) are presented in Table 3. HS-Raw had a low surface area (1.68 m^2^/g), whereas all pyrolyzed samples showed markedly larger surface areas. This increase is consistent with the development of a porous carbon structure as volatile components are released during pyrolysis [28].

Among the temperature-series biochars, HS-900 had the largest BET surface area, average pore diameter, and pore volume. HS-500 had a larger surface area than HS-700 but a much smaller average pore diameter and lower pore volume, indicating a greater contribution from narrow pores. These trends are consistent with the strong dependence of biochar pore structure on feedstock and pyrolysis temperature [52,53].

For the residence-time comparison at 700 °C, the standard 2 h sample had a higher BET surface area (381.94 versus 310.77 m^2^/g) and pore volume (0.2185 versus 0.1698 cm^3^/g) than HS-700 (1 h). Under the tested conditions, the longer residence time was therefore associated with more extensive physical pore development.

Changing the heating rate from 10 to 5 °C/min produced comparatively modest differences in BET surface area, pore size, and pore volume under the tested one-factor comparison. Decreasing the initial particle size to 5–10 mm was associated with lower surface area and pore volume than the standard 15–20 mm condition. Direct use of the 15–20 mm raw material also avoids an additional milling step before pyrolysis.

The BET surface areas and pore structures of the biochars varied primarily with pyrolysis temperature. The surface area increased from 1.68 m^2^/g for HS-Raw to 666.05 m^2^/g for HS-900. HS-500 also had a high surface area (484.92 m^2^/g), but its low pore volume was consistent with a large contribution from narrow pores. HS-700 had a surface area of 381.94 m^2^/g with intermediate pore volume and average pore diameter. Surface area alone is insufficient to predict oil-purification performance; pore architecture and surface chemistry must also be considered. Pore architecture may affect the accessibility of oil-degradation products, while surface functional groups can influence interactions with polar compounds [45,54]. Frying-oil degradation produces chemically diverse primary and secondary oxidation products [55]. HS-700 and HS-900 therefore appeared structurally promising as adsorbents, although the loss of oxygen-containing surface groups at high temperature can modify adsorption mechanisms [52,54]. The BET results were consequently interpreted together with SEM, FT-IR, and oil-purification performance rather than as a stand-alone ranking.

### 3.5. Fourier Transform Infrared Spectroscopy (FT-IR)

**Figure 3:**
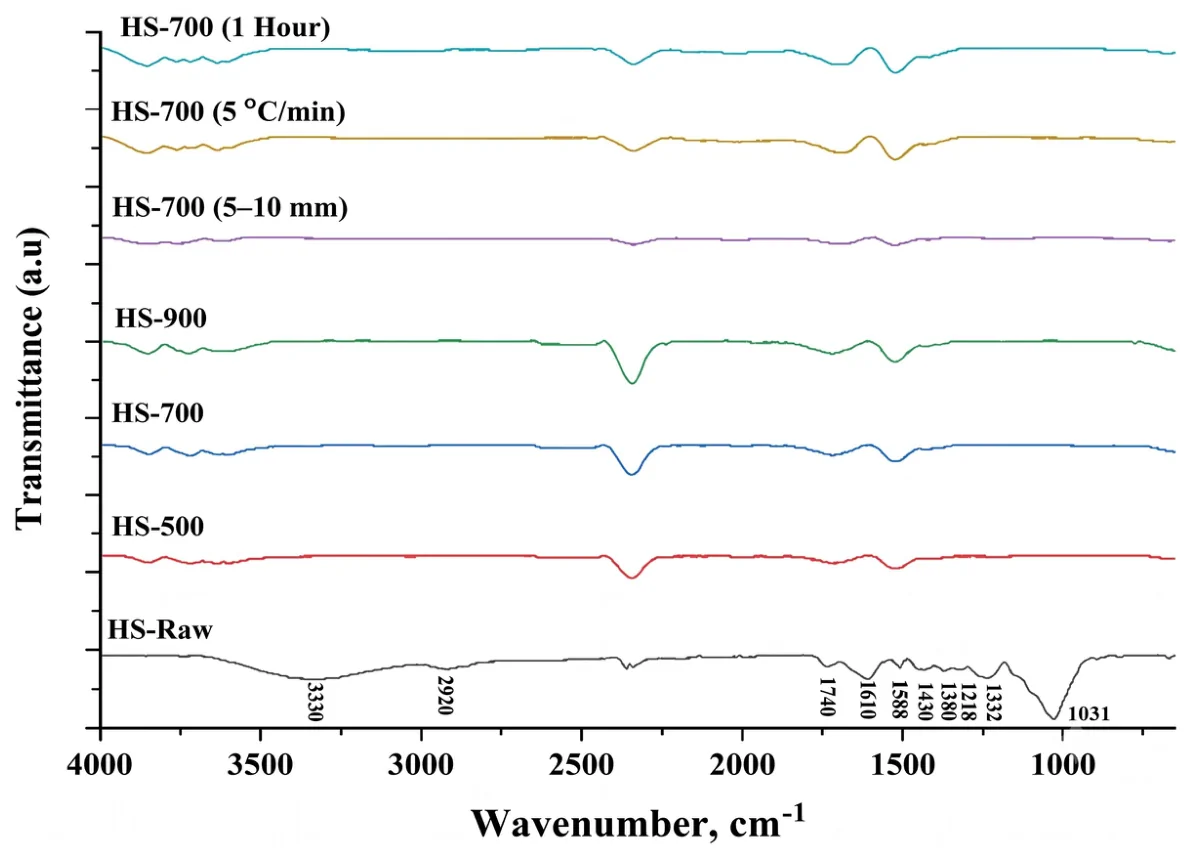
FT-IR spectra of raw hazelnut shells (HS-Raw) and biochar samples produced under different pyrolysis conditions (HS-500, HS-700, HS-900, HS-700 (5–10 mm), HS-700 (5 °C/min), and HS-700 (1 h)). The principal absorption bands identified for HS-Raw were located at approximately 3330, 2920, 1740, 1610, 1508, 1421, 1381, 1232, and 1031 cm^−1^.

FT-IR spectroscopy was used to assess changes in functional groups during pyrolysis (Figure 3). HS-Raw exhibited a broad O–H band near 3330 cm^−1^ and an aliphatic C–H/CH_2_ stretching band near 2920 cm^−1^, consistent with the cellulose–hemicellulose–lignin matrix. These bands were markedly attenuated from 500 °C onward, indicating progressive dehydration, devolatilization, and aromatic condensation [52,56]. The attenuation of O–H and aliphatic C–H bands is consistent with reduced surface polarity, but adsorption performance cannot be inferred from these bands alone.

HS-Raw showed a distinct C=O band near 1740 cm^−1^, attributable to carbonyl-containing species such as esters and carboxylic acids, which markedly diminished after pyrolysis. Bands in the 1610–1508 cm^−1^ region were assigned to aromatic C=C vibrations. Their broadening and reduced definition in the biochars, particularly HS-900, are consistent with the condensation of discrete aromatic and aliphatic moieties into more highly fused carbon domains, which commonly produce broad and weak IR absorption [52,56]. Bands near 1420 and 1380 cm^−1^, assigned to CH_2_ scissoring and CH_3_ bending vibrations, respectively, were also strongly attenuated, supporting the transition from an aliphatic-rich biomass matrix toward a more condensed carbonaceous structure.

The C–O stretching region associated with cellulose and hemicellulose (approximately 1232–1006 cm^−1^) was markedly attenuated in the biochars. This indicates substantial loss of polysaccharide-related C–O functionality during pyrolysis; it does not imply the complete absence of oxygen-containing structures, as also shown by the EDS results.

In used-oil purification, the marked reduction in O–H and aliphatic C–H bands indicates progressive deoxygenation and carbonization of the biochar surface. These changes may reduce surface polarity and modify interactions with oil-phase constituents. Biochars produced at 700 °C and above exhibited markedly reduced oxygen-containing functional groups and a well-developed porous structure; however, their adsorption performance should be interpreted alongside the BET and oil-purification results rather than inferred from FT-IR alone.

In addition to temperature, particle size, residence time, and heating rate were examined. Changing the initial particle size from 15–20 to 5–10 mm did not produce a marked difference in the FT-IR spectra of the 700 °C biochars, suggesting broadly similar functional-group transformations within these size ranges.

Reducing the heating rate to 5 °C/min produced only modest spectral differences, including slightly weaker absorption in the carbonyl region near 1700 cm^−1^. This pattern may reflect more gradual volatile release and thermal transformation at the lower heating rate.

No marked differences in functional-group pattern were observed between HS-700 (1 h) and the standard HS-700 sample (2 h) within the resolution of the FT-IR spectra. Most detectable functional-group transformations at 700 °C therefore appeared to occur within the first hour, whereas the BET results indicate that physical pore development continued with longer residence time.

### 3.6. Morphological and Elemental Analyses

#### 3.6.1. Results of Scanning Electron Microscope (SEM)

**Figure 4:**
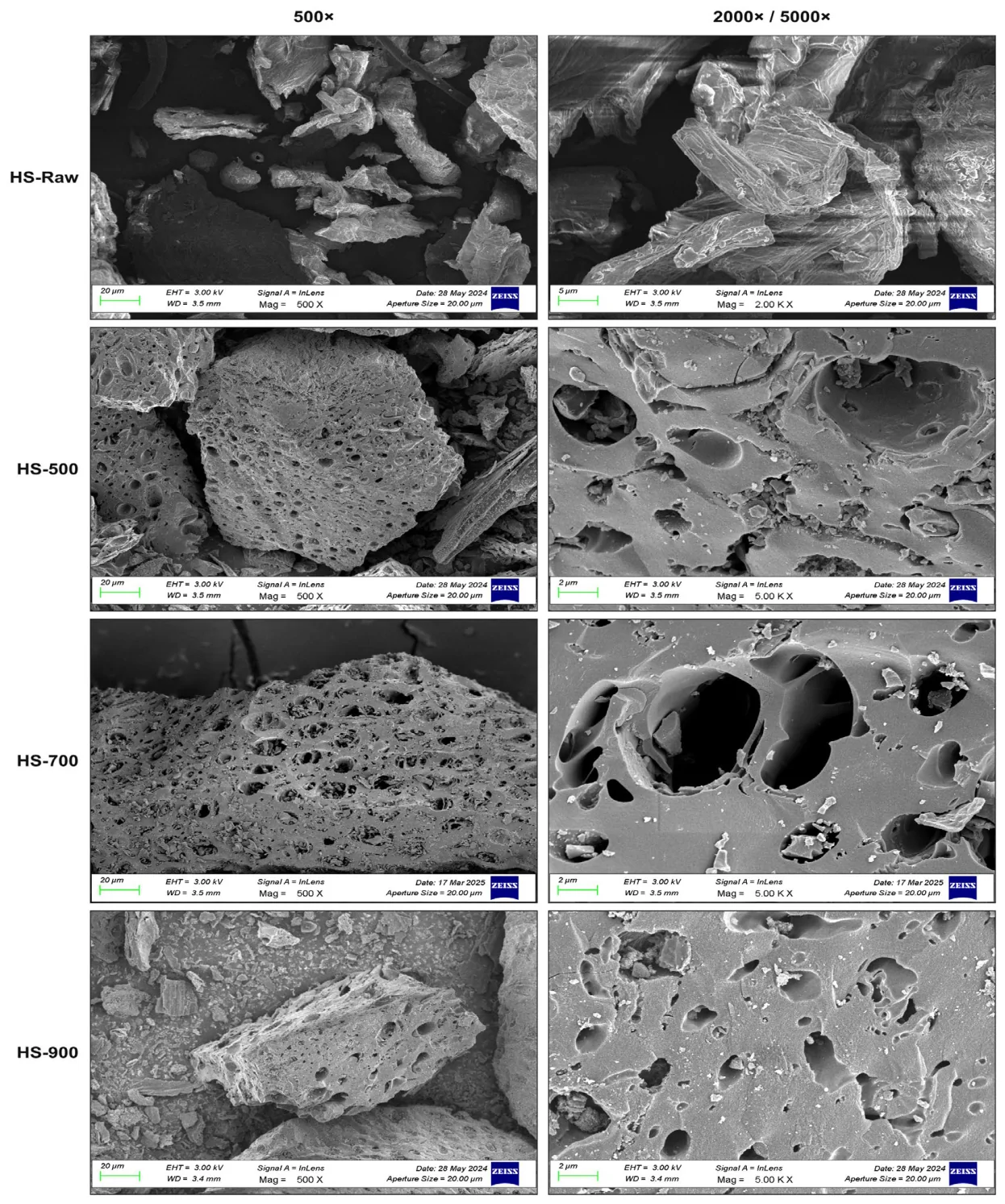
SEM micrographs of raw hazelnut shell (HS-Raw) and biochars produced at different pyrolysis temperatures. Low-magnification images were acquired at 500× with 20 µm scale bars. High-magnification images were acquired at 5000× with 2 µm scale bars, except for HS-Raw, which was acquired at 2000× with a 5 µm scale bar.

**Figure 5:**
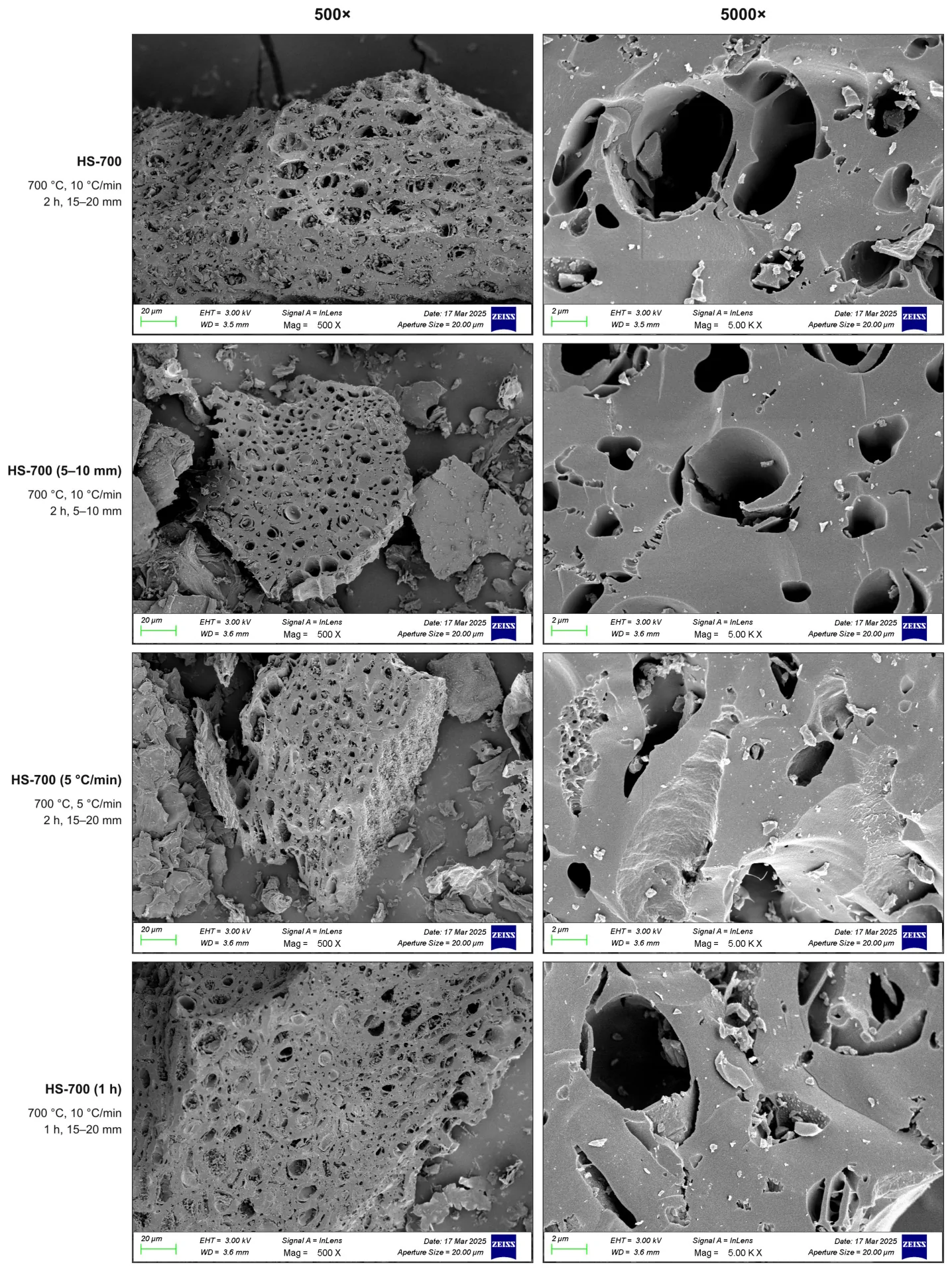
SEM micrographs of HS-700 biochars produced under different particle-size, heating-rate, and residence-time conditions. Low- and high-magnification images were acquired at 500× with 20 µm scale bars and at 5000× with 2 µm scale bars, respectively.

SEM micrographs acquired at 500× and 5000× magnification (2000× for HS-Raw) are presented in Figure 4 and Figure 5. HS-Raw exhibited a relatively dense and layered surface with limited visible porosity, reflecting the retained lignocellulosic structure. In contrast, all biochar samples showed cracks and open cavities generated during pyrolysis. These hollow, honeycomb-like features can be attributed to the removal of organic constituents from the original vascular tissue. Thus, the term “vascular channels” refers to the preserved anatomical pathways of the hazelnut shell rather than newly formed vein-like bundles. Increasing pyrolysis temperature generally promoted cracking and pore opening; however, SEM observations are qualitative and local and should not be interpreted as direct pore-size measurements.

For the HS-700 (5–10 mm) sample, the SEM micrographs showed a relatively uniform distribution of open cavities and comparatively smooth pore walls. The smaller initial particle size may have facilitated more uniform heat transfer and volatile release during pyrolysis. However, because SEM provides localized and qualitative observations, differences in pore size and connectivity should be interpreted together with the BET results.

The HS-700 (5 °C/min) sample exhibited a relatively regular porous matrix with smoother and apparently thicker pore walls. The lower heating rate may have allowed more gradual heat transfer and volatile release, thereby limiting abrupt structural disruption. Nevertheless, these morphological observations are qualitative and do not independently demonstrate differences in pore size.

The HS-700 (1 h) sample retained a porous structure, although some cavities appeared partially obstructed by residual surface material. Compared with the sample produced using a 2 h residence time, the shorter residence time may have provided less opportunity for further devolatilization and pore opening. However, SEM alone cannot identify the residual material as tar or conclusively demonstrate incomplete carbonization.

Taken together, the SEM observations, biochar yield, and BET results indicate that HS-700 produced under the reference conditions provided a practical balance between porous-structure development and process severity. Nevertheless, this selection should be regarded as condition-specific rather than as a fully optimized production condition.

#### 3.6.2. Energy-Dispersive X-Ray Spectroscopy (EDS)

The EDS results are summarized in Table 4, and the detailed reports are provided in Figures S1–S7. Because one representative spot was analyzed per sample, the values are localized and semi-quantitative. Relative carbon increased from 84.8 wt.% in HS-500 to 92.6 wt.% in HS-700, then decreased to 84.6 wt.% in HS-900 as the relative oxygen and mineral fractions increased. The HS-900 result may reflect localized heterogeneity and mineral enrichment and should not be interpreted as a bulk carbon mass balance [47,52].

Compared with the standard HS-700 sample, HS-700 (5–10 mm) showed lower localized carbon and higher oxygen and potassium percentages. Faster heat transfer in smaller particles may alter volatile release and local surface chemistry; however, the single-spot EDS measurements do not establish secondary gasification or bulk elemental loss.

The larger geometric surface area of smaller particles can also increase vapor–solid interactions during pyrolysis. Recondensation or chemisorption of oxygen-containing volatile species may therefore have contributed to the higher localized oxygen percentage in HS-700 (5–10 mm) than in the standard HS-700 sample [51]. This interpretation remains tentative because EDS was not spatially replicated.

Reducing the heating rate from 10 to 5 °C/min produced a localized EDS composition broadly similar to that of the standard HS-700 sample, consistent with comparable overall carbonization at the same final temperature.

HS-700 (1 h) had the highest localized carbon percentage among the 700 °C variants. This may indicate greater carbon retention after the shorter residence time; however, because the EDS results represent single locations, the difference should not be interpreted as proof of bulk carbon volatilization during the second hour.

The lower O/C atomic ratios of the biochars relative to HS-Raw are consistent with progressive deoxygenation, reduced surface polarity, and increased hydrophobicity during pyrolysis [57]. These changes may favor interaction with the oil phase, although oil-purification performance also depends on pore accessibility and the chemistry of the target compounds.

Potassium and calcium detected by EDS are minerals naturally present in hazelnut shells. If present in accessible ionic forms, such cations could contribute to adsorption through bridging interactions with polar carboxylate-containing species [58]; their actual chemical form and contribution were not determined here.

#### 3.6.3. ICP-OES Elemental Profiles of the Raw Material and Biochar Samples

The acid-extractable elemental concentrations determined by ICP-OES are presented in Table 5. Among the quantified elements, Ca and K occurred at the highest concentrations. Within the biochar series, Ca increased from HS-500 to HS-900, whereas K increased from HS-500 to HS-700 and then decreased slightly in HS-900. These variations are consistent with changes in the acid-extractable mineral fraction under different pyrolysis conditions. The HS-Raw values are included as a compositional reference; however, because USEPA Method 3051A is not a total-digestion procedure and extraction recovery may differ between lignocellulosic and carbonized matrices, the results were not used to calculate elemental retention or for direct mass-balance comparison with the gravimetric ash content.

The relatively high acid-extractable Ca concentrations do not establish the oxidation state or chemical form of Ca in the biochars. Any contribution of Ca-mediated bridging to the adsorption of polar oil-degradation products therefore remains a mechanistic hypothesis.

For descriptive screening, the acid-extractable concentrations were compared with OIV specifications for oenological bentonite [59] and EBC-FeedPlus reference values [60]. These frameworks address different applications and prescribe analytical methods that are not equivalent to the adapted USEPA 3051A extraction used here; the comparison therefore cannot establish regulatory compliance or food-contact suitability. Most measured concentrations did not exceed the corresponding numerical reference values. However, Cd compliance could not be assessed because the LOQ (1.00 mg/kg) exceeded the EBC-FeedPlus value (0.8 mg/kg), and the As concentration in HS-900 (1.99 mg/kg) was close to the 2 mg/kg reference value. A food-contact assessment would additionally require migration testing and analysis of process-related contaminants such as polycyclic aromatic hydrocarbons (PAHs).

**Table 5:** Acid-extractable elemental concentrations of the raw material and biochar samples after an adapted USEPA Method 3051A extraction, determined by ICP-OES (mg/kg, dry-weight basis).

Element (mg/kg dw)	HS-Raw	HS-500	HS-700	HS-900	EBC-FeedPlus ^1^	Bentonite ^2^
Cd	<1.00	<1.00	<1.00	<1.00	0.8	–
Cr	13.78	1.40	33.31	30.04	70	–
Co	<1.00	<1.00	<1.00	<1.00	–	–
Cu	16.47	9.48	19.75	44.31	70	–
Ni	5.69	1.20	12.27	12.38	25	–
Pb	2.89	<1.00	2.09	1.70	10	5
Zn	16.77	<1.00	15.36	13.57	200	–
Al	78.46	40.93	58.45	113.77	–	2500
As	<1.00	<1.00	<1.00	1.99	2	2
Ca	7087.24	5091.85	9076.40	11,077.84	–	–
Fe	179.68	50.92	299.22	369.26	–	600
K	9782.39	8087.06	15,459.80	14,071.86	–	–
Mn	105.81	73.88	109.71	155.69	–	–
P	344.38	285.54	613.41	718.56	–	–

^1^ European Biochar Certificate (EBC) FeedPlus reference values [60]. ^2^ OIV bentonite specification values [59]. dw, dry weight; LOQ, limit of quantification. Values are single determinations; concentrations below the LOQ are reported as <1.00 mg/kg. The analytical procedures prescribed by the reference frameworks differ from the adapted USEPA 3051A extraction used in this study; the numerical comparison is therefore preliminary and does not establish compliance. Cd compliance could not be assessed because the LOQ (1.00 mg/kg) exceeded the EBC-FeedPlus limit value (0.5 mg/kg). The As concentration in HS-900 (1.99 mg/kg) was close to the corresponding reference value (2 mg/kg) and requires confirmatory analysis.

### 3.7. Multivariate Correlation Assessment

**Figure 6:**
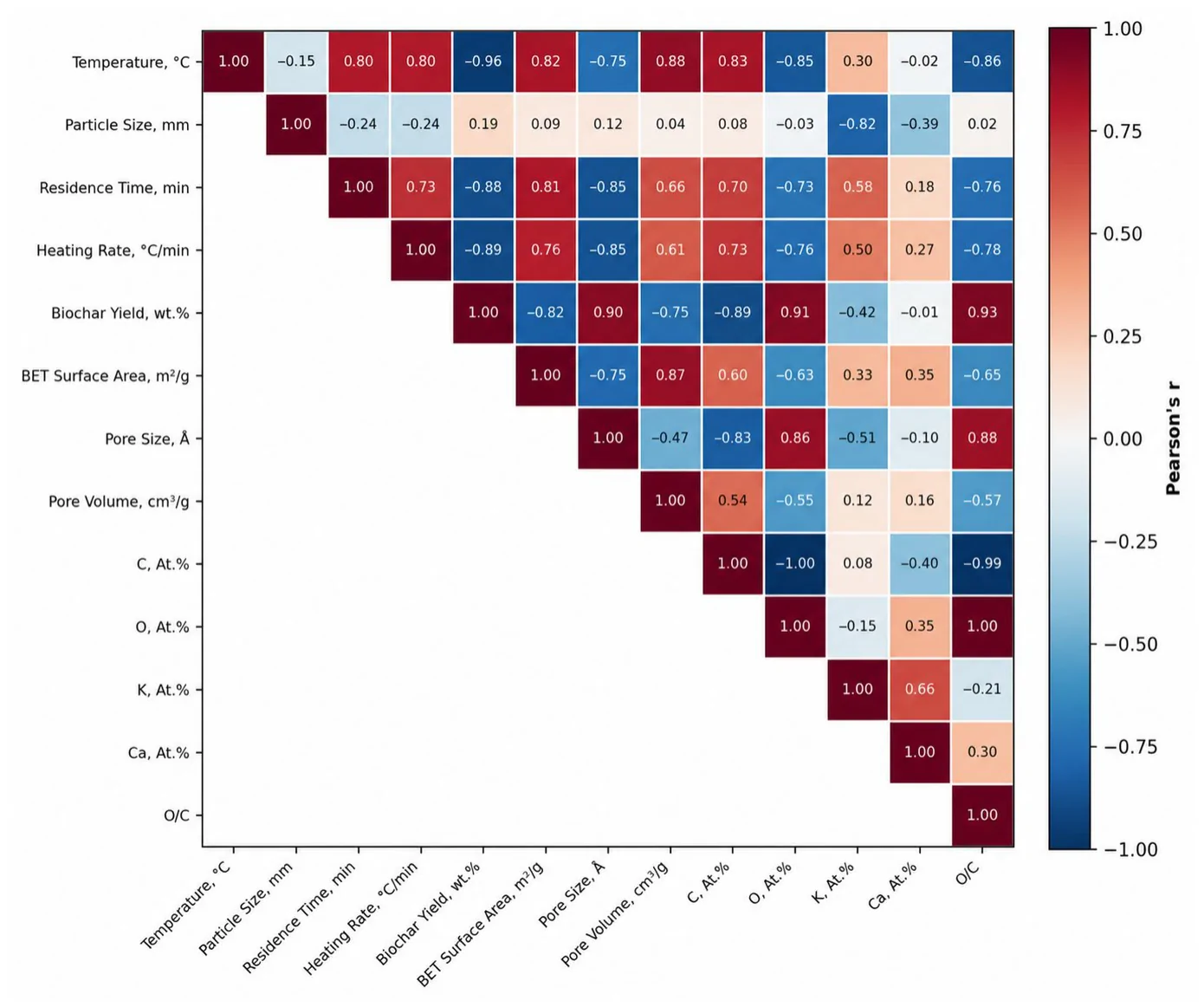
Pearson correlation matrix heatmap showing the relationships among pyrolysis conditions (temperature, particle size, residence time, and heating rate), biochar yield, BET surface area, pore size, pore volume, elemental composition (C, O, K, and Ca; at.%), and the O/C atomic ratio. Cell values represent Pearson’s correlation coefficients (r); red and blue indicate positive and negative correlations, respectively, and color intensity reflects the magnitude of the relationship. The corresponding significance levels are provided in Table S1.

A Pearson correlation matrix was constructed to comprehensively evaluate the system dynamics of the hazelnut shell pyrolysis process through the linear relationships between process independent variables (temperature, particle size, residence time, and heating rate) and product dependent variables (biochar yield, BET surface area, pore size, pore volume, atomic C, O, K, and Ca content, and O/C ratio). Pearson’s correlation coefficients for all pairwise relationships are presented in Figure 6, while the corresponding significance levels are provided in Table S1.

Pyrolysis temperature showed a strong negative correlation with biochar yield (r = −0.96, two-tailed *p* ≈ 0.0006). This was the only pairwise association that met the Bonferroni-corrected threshold (α ≈ 0.00064), and it is consistent with greater devolatilization and lower solid yield as pyrolysis severity increases [47,50].

Temperature showed nominal positive correlation with atomic carbon content (r = 0.83, uncorrected *p* < 0.05) and nominal negative correlations with atomic oxygen content (r = −0.85, uncorrected *p* < 0.05) and the O/C ratio (r = −0.86, uncorrected *p* < 0.05). These associations did not survive Bonferroni correction and are therefore interpreted as exploratory trends consistent with dehydration and deoxygenation at higher temperatures [46,47].

Temperature also showed nominal positive correlations with BET surface area (r = 0.82, uncorrected *p* < 0.05) and pore volume (r = 0.88, uncorrected *p* < 0.01). Although neither association met the Bonferroni-corrected threshold, their direction is consistent with pore development as volatile matter leaves the solid matrix.

The positive pairwise correlations of residence time and heating rate with temperature (r = 0.80 in each case) largely reflect the experimental design and the contrast between the unpyrolyzed raw material and the pyrolysis series.

At the uncorrected level, residence time and heating rate were negatively correlated with biochar yield (r = −0.88 and −0.89, respectively; *p* < 0.01) and positively correlated with BET surface area (r = 0.81 and 0.76, respectively; *p* < 0.05). Pore size was negatively correlated with both residence time and heating rate (r = −0.85 in each case; uncorrected *p* < 0.05). None of these associations met the Bonferroni-corrected threshold, and the process variables were not independently varied in a factorial design; the relationships should therefore be regarded as exploratory. Their directions may reflect secondary vapor–char reactions and progressive pore opening under more severe conditions [51,61]. Biochar yield was negatively correlated with BET surface area (r = −0.82, uncorrected *p* < 0.05) and positively correlated with pore size (r = 0.90, uncorrected *p* < 0.01). The association between BET surface area and pore diameter was negative but borderline at the uncorrected level (r = −0.75, *p* ≈ 0.052). Pore volume correlated positively with BET surface area (r = 0.87, uncorrected *p* < 0.05) but not significantly with any single process variable. These patterns are consistent with coupled changes in surface area and pore architecture, but they do not establish a causal mechanism [57,62].

Biochar yield showed nominal positive correlations with oxygen content (r = 0.91, uncorrected *p* < 0.01) and the O/C atomic ratio (r = 0.93, uncorrected *p* < 0.01), and a nominal negative correlation with carbon content (r = −0.89, uncorrected *p* < 0.01). None met the Bonferroni-corrected threshold. Nevertheless, their directions are consistent with the progressive conversion of an oxygen-rich lignocellulosic matrix into a more condensed, carbon-rich structure as pyrolysis severity increases [52,56,62].

### 3.8. Principal Component Analysis (PCA)

**Figure 7:**
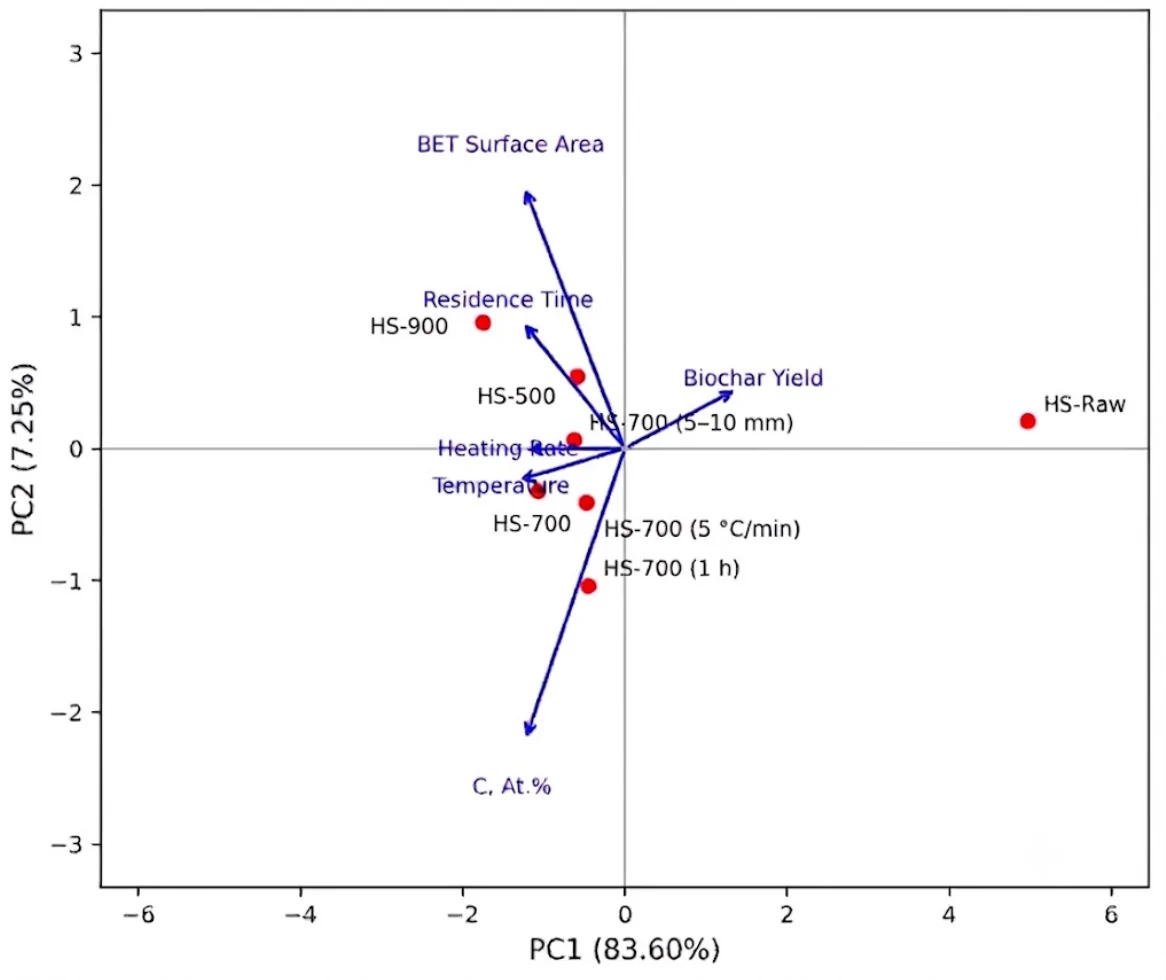
Biplot of the principal component analysis performed on the pyrolysis process and characterization variables (temperature, residence time, heating rate, biochar yield, BET surface area, and carbon content) for the seven samples.

Principal component analysis (PCA) was used to visualize joint variation in the selected pyrolysis-process and characterization variables. The two-dimensional biplot is shown in Figure 7, and total variance and factor loadings are reported in Tables S2 and S3, respectively. PC1 and PC2 together explained 90.85% of the variance (83.60% and 7.25%, respectively). PC2 had an eigenvalue below the traditional Kaiser threshold (λ = 0.43) but was retained for descriptive visualization because it increased cumulative explained variance above 80%. Given the small, systematically designed sample (n = 7) and the design-confounding and multiple-comparison limitations noted in Section 2.8, the PCA is interpreted as exploratory and hypothesis-generating rather than as a validated inferential model.

PC1 represents the macro-level axis of thermal decomposition and carbonization intensity. In Figure 7, HS-Raw is positioned on the positive side of PC1 and aligns with the biochar-yield vector, whereas the thermally treated biochar samples cluster on the negative side, toward the vectors representing temperature, carbon content, and BET surface area. Because the sign of a PCA axis is arbitrary, this orientation does not alter the interpretation: movement from HS-Raw toward the negative PC1 direction represents increasing pyrolysis severity, accompanied by lower biochar yield, greater carbon enrichment, and increased surface-area development.

In the exploratory score plot, PC2 separated some of the microstructural and chemical variation among the biochar samples, particularly within the HS-700 process variants. HS-900 was located in the upper part of PC2 and oriented toward the BET-surface-area vector, consistent with its measured maximum surface area. Because PC2 explained only 7.25% of the variance and had an eigenvalue below 1, this separation should be interpreted descriptively.

HS-700 (1 h) was located in the lower-left quadrant and aligned most closely with the atomic-carbon vector. Within this small, systematically designed dataset, the pattern is consistent with greater localized carbon retention after the shorter residence time, whereas the 2 h condition had higher BET surface area and pore volume. It does not demonstrate that either residence time is globally optimal.

Taken together, the Pearson matrix and PCA provide an exploratory framework for describing the hazelnut-shell biochars produced under the tested conditions. HS-900 had the highest BET surface area, which may increase access to adsorptive pore space, whereas HS-700 (1 h) had the highest localized carbon percentage among the 700 °C variants [52,54]. Potassium and calcium may contribute to surface interactions with polar degradation products if present in accessible ionic forms [58]; conversely, migration of mineral species into oil could be undesirable. Because only acid-extractable concentrations in the biochars were measured (Section 3.6), neither the adsorption contribution nor migration of these elements was established. The residence-time, heating-rate, and particle-size variants were not evaluated in the oil-purification experiments, which used only HS-500, HS-700, and HS-900. The chemometric results therefore generate hypotheses for future experiments rather than a validated ranking of purification performance.

### 3.9. Waste Frying Oil Purification Performance of Hazelnut Shell Biochars

The three hazelnut shell biochars were evaluated as adsorbents for the purification of waste frying oil and were benchmarked against two commercial adsorbents (activated carbon and Magnesol). Purification performance was assessed through a set of complementary oxidative and hydrolytic quality indicators, and the results are discussed below in relation to the physicochemical properties of the biochars established in the preceding sections.

#### 3.9.1. Free Fatty Acidity (FFA) and Total Polar Material (TPM)

Free fatty acidity reflects the accumulation of free fatty acids released through triacylglycerol hydrolysis, whereas total polar material represents the sum of oxidation and polymerization products; TPM is regarded as the most reliable overall indicator for determining the useful life of a frying oil, with values above 25% indicating that the oil should be discarded [11]. These two parameters, therefore, constitute the most direct measures of adsorbent purification efficiency. The FFA and TPM results are summarized in Table 6.

One-way analysis of variance revealed a highly significant difference among the treatment groups for FFA (F(5, 12) = 31.5, *p* < 0.001). All adsorbents significantly reduced the free fatty acid content relative to the control (reductions of 37.8% for HS-500, 27.6% for HS-700, 34.6% for HS-900, and 33.3% for AC). According to the Tukey test, the three biochars and the commercial activated carbon fell within the same statistical group (b), indicating that no statistically significant difference was detected between the FFA removal performance of hazelnut shell biochars and that of commercial activated carbon under the tested conditions. Magnesol alone achieved the highest reduction (55.8%) and was assigned to a separate group (c). This finding is consistent with reports that magnesium silicate–based adsorbents exhibit high affinity for free fatty acids through Lewis acid–base and polar interactions at their surfaces; in a packed-column study, alumina and Magnesol improved the free fatty acid content by 103% and 105%, respectively, while silica gel improved total polar material by 100% [18].

For TPM, one-way ANOVA among all six groups (WFO and the five adsorbent-treated samples) showed no statistically significant differences (F(5, 12) = 0.48, *p* = 0.785). All groups fell within a single Tukey grouping (a), indicating that none of the adsorbents produced a statistically detectable TPM reduction under the tested conditions (60 °C, 15 min contact, 4% *w*/*w*). This contrasts with the pronounced FFA reductions and is consistent with the absence of significant changes in K_232_ and K_268_ (Section 3.9.2). The removal of polar degradation products under more intensive conditions is well documented: silica gel showed high adsorption capacity for total polar material in used frying oil [42], while binary and quaternary adsorbent mixtures and multi-pass packed-column systems were more effective than single-stage treatment [16]. The present result therefore suggests that longer contact times or column operation may be required for a measurable TPM improvement.

The differences in FFA and AnV between the untreated oil and the adsorbent-treated samples were statistically significant according to the respective one-way ANOVA results (*p* < 0.001). Tukey HSD comparisons confirmed that all adsorbent-treated groups had lower FFA and AnV values than the untreated control (*p* < 0.05). In contrast, TPM values did not differ significantly among the groups (*p* = 0.785), consistent with the mild, short-contact batch conditions applied.

The adsorption conditions used here (60 °C, 15 min, 4% *w*/*w*) were selected to represent a warm, short-contact treatment and to provide a standardized comparison with the commercial adsorbents rather than to optimize the process. Systematic optimization of contact time, temperature, and dose remains necessary. The significant FFA and AnV reductions indicate that hazelnut-shell biochars can remove selected degradation products during short batch contact, whereas their ability to reduce the broader TPM pool was limited under these conditions. These findings support further evaluation of waste-derived hazelnut-shell biochar as a circular adsorbent, particularly for applications targeting free fatty acids and secondary oxidation products.

The reduction in FFA is also relevant to potential downstream valorization because a low FFA content is required for efficient base-catalyzed transesterification. Adsorbent pretreatment of waste frying oil has been reported to improve feedstock quality and the yield and oxidative stability of the resulting biodiesel [63]. Agricultural-waste adsorbents have also been used downstream in the biodiesel chain; durian-peel adsorbents, for example, have been applied to the purification of acidified crude glycerol [64]. Because TPM and the broader polymerized fraction were not significantly reduced under the mild batch conditions used here, additional pretreatment would still be required before evaluating the treated oil as a biodiesel feedstock.

As summarized in Table 7, the FFA removal achieved by the hazelnut-shell biochars (27.6–37.8%) under mild, single-stage batch conditions was lower than the higher removals reported for chemically activated carbons or packed-column systems (e.g., 56% FFA removal, together with a 92% reduction in p-anisidine value, for apricot-kernel activated carbon in a packed column [22], and 92.8% FFA removal for microwave–KOH-activated durian-peel carbon [65]). Most higher-efficiency results in the literature were obtained with chemical or physical activation or with longer/multi-pass treatment, whereas the present biochars were unactivated and used in a single 15 min batch contact. Some activated carbons also markedly lighten oil color; by contrast, the hazelnut-shell biochars produced only small color changes (ΔE* < 2) and did not darken the oil. The comparison supports the practical FFA and AnV performance of the unactivated biochars while indicating that activation or column operation may be needed to improve TPM removal.

#### 3.9.2. Peroxide Value (PV) and Specific Extinction Coefficients (K_232_–K_268_)

The peroxide value measures hydroperoxides formed during the primary stage of oxidation, whereas K_232_ and K_268_ quantify the UV absorption of conjugated diene and triene structures, respectively [72]. The corresponding PV, K_232_, and K_268_; results are presented in Table 8.

Analysis of variance indicated a significant difference among groups for PV (F(5, 12) = 76.8, *p* < 0.001). The Tukey test showed that the hazelnut-shell biochars did not lower PV and remained in the same group as the control (a), whereas activated carbon produced a limited reduction (1.9%; b) and Magnesol a larger reduction (12.8%; c). Hydroperoxides are transient primary oxidation products that can both form and decompose during heating [55]. The absence of a net PV decrease after biochar treatment may therefore reflect the combined effects of adsorption, hydroperoxide formation or decomposition during the 60 °C treatment, and the short contact time. The stronger response to Magnesol is an empirical result of the present experiment; the specific surface interactions responsible were not directly measured.

No statistically significant differences were found among the groups for either K_232_ or K_268_ (*p* = 0.469 and *p* = 0.278, respectively). Thus, the treatments did not produce a detectable change in the conjugated diene and triene indicators under the single-stage, short-duration batch conditions. Larger effects have been reported with more intensive systems; for example, silica gel improved conjugated-diene content by 84% in a multi-pass packed-column treatment [18]. The present lack of significant change may therefore reflect the treatment mode and duration together with the variability of these measurements; selective retention of the corresponding compounds was not measured directly.

#### 3.9.3. p-Anisidine Value (AnV)

The p-anisidine value measures carbonyl secondary oxidation products formed during hydroperoxide decomposition, particularly 2-alkenals and 2,4-alkadienals [73]. In this study, AnV decreased significantly in all adsorbent-treated groups relative to the control, consistent with removal of secondary carbonyl products. The p-anisidine values of all treatment groups are presented in Table 9.

One-way analysis of variance revealed a highly significant difference among the six groups (F(5, 12) = 70.6, *p* < 0.001). The untreated control formed a distinct higher Tukey group (a), whereas all five adsorbent-treated samples formed one lower group (b), corresponding to reductions of 22.3–27.5%. No statistically significant difference was detected among the biochars and the two commercial adsorbents. The consistent relative decrease supports a treatment-related reduction in the secondary carbonyl products measured by the p-anisidine assay [73].

#### 3.9.4. Color Characteristics (L*, a*, b*, and ΔE*)

The color of a frying oil is an important quality attribute that can change due to the accumulation of degradation products and to the bleaching effect of adsorbents. The color parameters and the calculated total color differences are presented in Table 10.

No significant differences were found among the groups for lightness (L*) or a* (*p* = 0.395 and *p* = 0.675, respectively). A significant difference was detected for b* (*p* = 0.006); HS-900 and Magnesol slightly reduced yellowness relative to the control. Nevertheless, the total color differences (ΔE*) ranged only from 0.71 to 1.82, indicating small instrumental changes relative to the already light-colored WFO.

The color results have two practical implications. First, treatment with black powdered biochar did not significantly reduce oil lightness (L*), indicating that centrifugation and filtration removed enough fine particles to avoid measurable darkening under the test conditions. Second, HS-900 and Magnesol produced a small reduction in yellowness (b*). Adsorbents can produce much larger color changes in darker or more degraded oils; for example, activated carbon and Magnesol improved color by 100% and 90%, respectively, in a packed-column study [18]. The modest changes observed here are consistent with the high initial lightness of the WFO (L* ≈ 96). Chlorophyll and carotenoid pigments were not quantified; oxidative changes were monitored using K_232_, K_268_, PV, and AnV, while overall color was evaluated using CIE L*a*b* parameters. Some adsorbents that lower acidity also have little effect on pigments; Al_2_O_3_, SiO_2_, and TiO_2_ nano-adsorbents, for example, reduced the acid value of crude canola oil while leaving its carotenoid and chlorophyll contents largely unchanged [20].

#### 3.9.5. Fatty Acid Composition

Fatty acid composition was measured in triplicate by GC-FID. Peak assignments were verified against the external FAME standard described in Section 2.11, and the corrected assignments are reported in Table 11.

The waste frying oil was dominated by linoleic acid (~47.5%) and oleic acid (~37%), consistent with a high-linoleic sunflower-type oil. No significant differences were detected among the six groups for oleic acid (*p* = 0.771), linoleic acid (*p* = 0.247), α-linolenic acid (*p* = 0.099), ΣMUFA (*p* = 0.817), or ΣPUFA (*p* = 0.255). Under the tested conditions, adsorption therefore did not measurably alter the relative composition of the major glyceride-bound unsaturated fatty acids [16].

Significant differences were confined to selected saturated-fatty-acid percentages and were driven mainly by Magnesol: palmitic acid and ΣSFA were slightly higher than in the other groups (*p* < 0.001), and stearic acid was slightly higher in the activated-carbon and Magnesol groups (*p* = 0.005). These shifts were below approximately one percentage point and may partly reflect relative-area normalization rather than selective fatty-acid removal. All three hazelnut-shell biochars remained statistically indistinguishable from the control for every reported fatty acid.

Taken together, the fatty-acid data indicate that hazelnut-shell biochar treatment did not significantly alter the relative composition of the major glyceride-bound fatty acids while improving selected oil-quality parameters.

#### 3.9.6. Multivariate Relationships Among Oil Quality Parameters

To explore whether the oil quality parameters measured above (Section 3.9.1, Section 3.9.2, Section 3.9.3 and Section 3.9.4) covary in a consistent pattern across treatments, an exploratory Pearson correlation analysis was performed using the full set of triplicate measurements for FFA, PV, K_232_, K_268_, AnV, and TPM across the six treatment groups (n = 18). Given the number of pairwise comparisons (15), a Bonferroni-corrected significance threshold (α = 0.05/15 ≈ 0.0033) is reported alongside the uncorrected *p*-values. The resulting Pearson correlation coefficients are presented in Table 12.

Using the replicate-level dataset (n = 18), FFA and AnV remained positively correlated after Bonferroni correction (r = 0.738, *p* < 0.001), indicating that both measures decreased in a coordinated manner across treatments and replicates. This pattern is consistent with a common response to adsorbent treatment, but correlation alone does not establish a shared adsorption mechanism. Two additional associations reached uncorrected significance but did not survive Bonferroni correction: FFA with PV (r = 0.512, *p* = 0.030) and PV with K_268_ (r = −0.533, *p* = 0.023). These are treated as exploratory observations. FFA and TPM were not significantly correlated (r = 0.282, *p* = 0.257), consistent with the finding that the broader TPM pool was not significantly reduced under the tested conditions.

**Figure 8:**
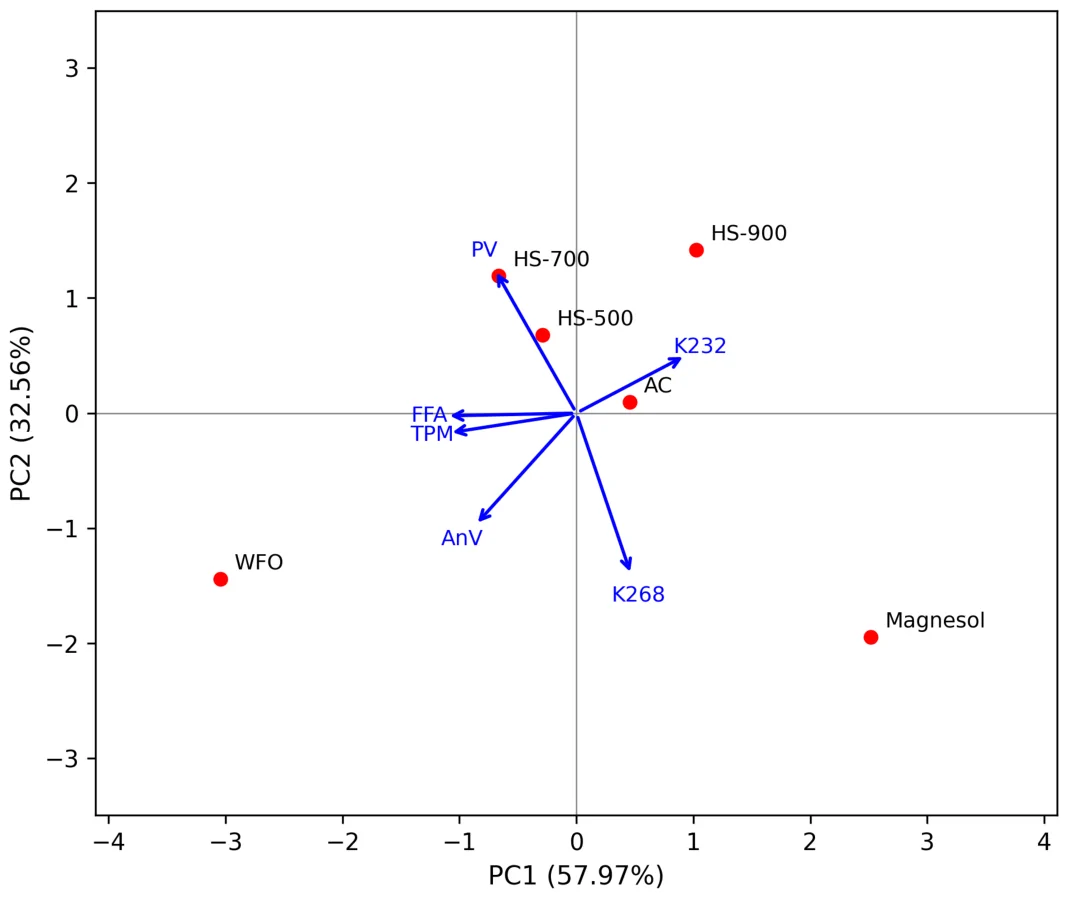
Biplot of the principal component analysis performed on the group-mean oil quality parameters (FFA, PV, K_232_, K_268_, AnV, and TPM) for the six treatment groups.

As a complementary visualization, an exploratory PCA was performed on the standardized group-mean values (n = 6) of the same six oil-quality parameters. The first two components together explained 90.54% of the variance (PC1 = 57.97%, PC2 = 32.56%). PC1 had negative loadings for FFA, TPM, and AnV and a positive loading for K_232_, producing a descriptive gradient from WFO on the negative side to Magnesol on the positive side, with the three biochars and activated carbon at intermediate positions (Figure 8). This biplot summarizes joint variation among the measured parameters but does not provide formal statistical separation among treatment groups.

## 4. Conclusions

This study demonstrated the conversion of hazelnut shells into carbon-based adsorbents and evaluated their performance in waste frying oil against two commercial benchmarks. The Pearson matrix and PCA provided an exploratory framework linking pyrolysis conditions with pore development and localized elemental composition; the first two principal components summarized 90.85% of the variance. Increasing final temperature markedly developed the pore structure, with a maximum BET surface area of 666.05 m^2^/g at 900 °C. FT-IR indicated that the major functional-group transformations at 700 °C were already established after 1 h, whereas the 2 h sample had higher BET surface area and pore volume, indicating more extensive physical pore development under the tested conditions.

In waste-frying-oil purification, all three hazelnut-shell biochars significantly reduced free fatty acidity (27.6–37.8%) and p-anisidine value (23.3–27.5%), with no statistically significant difference from commercial activated carbon under the tested conditions (Tukey HSD, *p* > 0.05). No statistically significant TPM reduction was detected under the mild, short-contact batch conditions. Magnesol produced the largest FFA and PV reductions. GC-FID analysis showed no significant changes in the relative composition of the major glyceride-bound unsaturated fatty acids after biochar treatment, and colorimetry showed no significant change in oil lightness.

The acid-extractable elemental concentrations provided a preliminary compositional comparison with reference values used for feed-grade biochar and oenological bentonite; they do not demonstrate regulatory compliance or food-contact suitability. Confirmation for oil-contact use will require analysis by the prescribed regulatory methods, migration testing, and determination of process-related contaminants. Overall, hazelnut-shell biochar is a promising waste-derived adsorbent for selected oil-degradation products and provides a potential circular valorization route for an important agricultural by-product in Türkiye. Future work should include process optimization, regeneration and reuse over multiple cycles, column-based purification, and a comprehensive safety assessment. Regeneration was not evaluated here, although reuse has been demonstrated for other oil-refining adsorbents; Al_2_O_3_ nano-adsorbents, for example, were reactivated by calcination and reused over three refining cycles [20].

## Figures and Tables

**Table 1:** Physicochemical properties of raw hazelnut shells.

Raw Material	Water Content (%)	Organic Matter (%dw)	Volatile Matter (%dw)	Ash (%dw)	Fixed Carbon (%dw)
Hazelnut Shell	7.80	98.90	80.06	1.10	18.84

Organic matter (%dw) was determined by loss-on-ignition (ASTM D2974-13) and is reported as 100 − Ash (%dw), consistent with the standard’s definition.

**Table 2:** Biochar yields at different pyrolysis temperatures and operating conditions.

Sample Code	Temp. (°C)	Particle Size (mm)	Residence Time (min)	Heating Rate (°C/min)	Biochar Yield (%)
HS-500	500	15–20	120	10	34.39
HS-700	700	15–20	120	10	26.31
HS-900	900	15–20	120	10	20.97
HS-700 (5–10 mm)	700	5–10	120	10	26.05
HS-700 (5 °C/min)	700	15–20	120	5	30.43
HS-700 (1 h)	700	15–20	60	10	28.71

**Table 3:** BET surface area and pore structure of the raw material and biochar samples.

Sample Code	BET Surface Area (m^2^/g)	Pore Size (Å)	Pore Volume (cm^3^/g)
HS-Raw	1.68	57.42	0.0024
HS-500	484.92	9.76	0.1183
HS-700	381.94	22.89	0.2185
HS-900	666.05	24.26	0.4040
HS-700 (5–10 mm)	318.79	21.48	0.1712
HS-700 (5 °C/min)	358.09	21.72	0.1944
HS-700 (1 h)	310.77	21.86	0.1698

Average pore diameter (D) was calculated using the single-point method: D = 4V/A, where V is the total pore volume, and A is the BET surface area.

**Table 4:** Localized, semi-quantitative EDS elemental composition (wt.% and at.%) of the raw material and biochar samples. One representative spot was analyzed per sample.

Sample	Basis	C	O	K	Ca	O/C
HS-Raw	wt.%	66.20	32.40	0.50	0.90	–
HS-Raw	at.%	72.79	26.74	0.17	0.30	0.37
HS-500	wt.%	84.80	11.10	2.50	1.60	–
HS-500	at.%	89.85	8.83	0.81	0.51	0.10
HS-700	wt.%	92.60	5.80	1.10	0.50	–
HS-700	at.%	95.03	4.47	0.35	0.15	0.05
HS-900	wt.%	84.60	11.90	1.60	1.60	–
HS-900	at.%	89.52	9.45	0.52	0.51	0.11
HS-700 (5–10 mm)	wt.%	82.10	12.50	3.60	1.50	–
HS-700 (5–10 mm)	at.%	88.24	10.09	1.19	0.48	0.11
HS-700 (5 °C/min)	wt.%	93.40	5.40	1.20	–	–
HS-700 (5 °C/min)	at.%	95.48	4.14	0.38	0.00	0.04
HS-700 (1 h)	wt.%	94.70	4.20	0.80	0.30	–
HS-700 (1 h)	at.%	96.45	3.21	0.25	0.09	0.03

**Table 6:** Effect of adsorbent treatments on the FFA and TPM of waste frying oil.

Parameter	WFO	HS-500	HS-700	HS-900	AC	Magnesol
FFA (%)	0.52 ± 0.03 ^a^	0.32 ± 0.05 ^b^	0.38 ± 0.02 ^b^	0.34 ± 0.03 ^b^	0.35 ± 0.01 ^b^	0.23 ± 0.03 ^c^
TPM (%)	36.00 ± 1.00 ^a^	35.67 ± 0.88 ^a^	35.56 ± 0.51 ^a^	35.33 ± 0.67 ^a^	35.67 ± 0.34 ^a^	35.22 ± 0.51 ^a^

WFO: waste frying oil (untreated control); HS-500, HS-700, HS-900: hazelnut shell biochars pyrolyzed at 500, 700, and 900 °C; AC: activated carbon; FFA: free fatty acidity; TPM: total polar material. The TPM of the untreated WFO was determined via a validated dilution series (Section 2.11). Different superscript letters within a row indicate statistically significant differences (Tukey HSD, *p* < 0.05). Percentage reductions were calculated from the unrounded group means.

**Table 7:** Comparison of the FFA and other quality-parameter removal efficiencies of various waste- and agricultural-derived, as well as commercial, adsorbents reported for used frying/cooking oils.

Adsorbent (Feedstock)	Oil Type	Conditions	FFA Removal	Other Key Results	Reference
Hazelnut shell biochar (HS-500/700/900)	WFO (sunflower)	60 °C, 15 min, 4% *w*/*w*, batch, no activation	27.6–37.8%	AnV reduced by 22.3–27.5%; no significant reduction in TPM; fatty-acid profile preserved; ΔE* < 2	This study
Nut-shell and apricot-kernel activated carbons	Used sunflower oil	Packed column	56%	TPM reduced by 100%, AnV by 92%, and color by 90% (apricot activated carbon)	Turan et al. [22]
Activated carbon, Fuller’s earth, MgO, silica gel, etc.	Sunflower, soybean, rice-bran, and palm-olein oils	150 °C, 30 min	Activated carbon most effective (palm olein)	Fuller’s earth reduced TPM in soybean oil; MgO reduced AnV in palm olein	Lisa et al. [66]
Sugarcane bagasse (NaOH-treated)	Frying oil	Active filtration	Significant reduction	FFA and TPM were reduced; oil life was extended	Ahmed et al. [67]
Durian peel activated carbon (KOH and microwave)	Used cooking oil	Optimized conditions; BET surface area: 400.68 m^2^/g	92.84%	PV reduced by 53.01%	Tambun et al. [65]
Sugarcane bagasse biochar-bentonite	Used cooking oil	10 g, 180 min	Not reported	PV reduced by 82.3–84.5%	Wijayanti et al. [68]
Palm kernel shell activated carbon (PKSAC)	Waste cooking oil	2.5% *w*/*w*	Decreased from 1.47% to 0.79%	PV decreased from 163 to 116 meq O_2_/kg; ΔE = 5.44 (color lightened)	Ulfah et al. [69]
Cocoa pod husk activated carbon	Used cooking oil	Batch; varied concentration and contact time	Reduced	Moisture content and PV were reduced	Arman et al. [70]
ZIF-8@biochar composite	Used frying oil	0.5% *w*/*w*	80.6%	PV reduced by 70.6%; acrylamide reduced by 99.9%	Abdelhameed et al. [24]
Zeolite and eggshell (natural adsorbents)	Used sunflower and palm oils	Batch	Reduced	FFA and PV were reduced; physicochemical properties improved	Bostan et al. [71]

Abbreviations: AC, activated carbon; AnV, p-anisidine value; FFA, free fatty acidity; PKSAC, palm kernel shell activated carbon; PV, peroxide value; TPM, total polar material; WFO, waste frying oil; ZIF-8, zeolitic imidazolate framework-8.

**Table 8:** Effect of adsorbent treatments on the PV, K_232_, and K_268_ of waste frying oil.

Parameter	WFO	HS-500	HS-700	HS-900	AC	Magnesol	ANOVA
PV (meq O_2_/kg)	43.6 ± 0.1 ^ab^	44.3 ± 0.9 ^a^	45.0 ± 0.4 ^a^	44.7 ± 0.6 ^a^	42.8 ± 0.4 ^b^	38.0 ± 0.3 ^c^	*p* < 0.001
K_232_	0.188 ± 0.006 ^a^	0.210 ± 0.032 ^a^	0.201 ± 0.017 ^a^	0.240 ± 0.051 ^a^	0.227 ± 0.008 ^a^	0.221 ± 0.048 ^a^	*p* = 0.469
K_268_	0.046 ± 0.001 ^a^	0.044 ± 0.002 ^a^	0.043 ± 0.003 ^a^	0.044 ± 0.001 ^a^	0.046 ± 0.005 ^a^	0.049 ± 0.005 ^a^	*p* = 0.278

Values are presented as mean ± SD (n = 3). Different superscript letters within the same row indicate significant differences among treatments according to Tukey’s HSD test (*p* < 0.05).

**Table 9:** Effect of adsorbent treatments on the p-anisidine value of waste frying oil.

Parameter	WFO	HS-500	HS-700	HS-900	AC	Magnesol	ANOVA
AnV	41.24 ± 1.05 ^a^	31.65 ± 0.68 ^b^	30.32 ± 1.05 ^b^	29.88 ± 0.94 ^b^	30.48 ± 1.02 ^b^	32.04 ± 0.41 ^b^	*p* < 0.001

Values are presented as mean ± SD (n = 3). Different superscript letters indicate significant differences among treatments according to Tukey’s HSD test (*p* < 0.05).

**Table 10:** Effect of adsorbent treatments on the color parameters of waste frying oil.

Parameter	WFO	HS-500	HS-700	HS-900	AC	Magnesol	ANOVA
L*	96.3 ± 1.22 ^a^	95.2 ± 0.63 ^a^	95.3 ± 1.26 ^a^	96.1 ± 1.02 ^a^	95.9 ± 0.15 ^a^	94.9 ± 0.77 ^a^	*p* = 0.395
a*	−2.99 ± 0.09 ^a^	−2.90 ± 0.04 ^a^	−2.90 ± 0.09 ^a^	−2.88 ± 0.10 ^a^	−2.90 ± 0.05 ^a^	−2.91 ± 0.09 ^a^	*p* = 0.675
b*	7.13 ± 0.20 ^a^	6.73 ± 0.14 ^ab^	6.49 ± 0.44 ^ab^	6.30 ± 0.32 ^b^	6.53 ± 0.10 ^ab^	5.95 ± 0.34 ^b^	*p* = 0.006
ΔE* (vs. WFO)	–	1.17	1.17	0.85	0.71	1.82	*–*

Values are presented as mean ± standard deviation (n = 3). Different superscript letters within a row indicate statistically significant differences according to Tukey’s HSD test (*p* < 0.05). ΔE* values were calculated from the corresponding mean L*, a*, and b* values relative to WFO.

**Table 11:** Fatty acid composition of waste frying oil and adsorbent-treated samples (relative area %, mean ± SD, n = 3).

Fatty Acid	WFO	HS-500	HS-700	HS-900	AC	Magnesol	ANOVA
Palmitic (C16:0)	10.16 ± 0.04 ^b^	10.02 ± 0.09 ^b^	10.00 ± 0.09 ^b^	10.02 ± 0.03 ^b^	10.23 ± 0.13 ^b^	10.80 ± 0.22 ^a^	*p* < 0.001
Stearic (C18:0)	3.56 ± 0.03 ^b^	3.58 ± 0.04 ^b^	3.59 ± 0.09 ^b^	3.56 ± 0.03 ^b^	3.64 ± 0.08 ^ab^	3.76 ± 0.02 ^a^	*p* = 0.005
Oleic (C18:1n9c)	37.33 ± 0.81 ^a^	37.14 ± 0.18 ^a^	37.02 ± 0.27 ^a^	37.15 ± 0.02 ^a^	37.03 ± 0.23 ^a^	36.88 ± 0.14 ^a^	*p* = 0.771
Linoleic (C18:2n6c)	47.38 ± 0.87 ^a^	47.72 ± 0.24 ^a^	47.66 ± 0.35 ^a^	47.67 ± 0.02 ^a^	47.54 ± 0.34 ^a^	46.91 ± 0.19 ^a^	*p* = 0.247
Linolenic (C18:3)	0.27 ± 0.01 ^a^	0.26 ± 0.01 ^a^	0.27 ± 0.00 ^a^	0.27 ± 0.01 ^a^	0.27 ± 0.00 ^a^	0.28 ± 0.00 ^a^	*p* = 0.099
ΣSFA	14.81 ± 0.06 ^b^	14.67 ± 0.10 ^b^	14.67 ± 0.16 ^b^	14.64 ± 0.04 ^b^	14.94 ± 0.23 ^b^	15.68 ± 0.22 ^a^	*p* < 0.001
ΣMUFA	37.54 ± 0.80 ^a^	37.35 ± 0.19 ^a^	37.40 ± 0.26 ^a^	37.42 ± 0.02 ^a^	37.25 ± 0.23 ^a^	37.13 ± 0.14 ^a^	*p* = 0.817
ΣPUFA	47.66 ± 0.86 ^a^	47.98 ± 0.24 ^a^	47.93 ± 0.35 ^a^	47.94 ± 0.03 ^a^	47.81 ± 0.34 ^a^	47.19 ± 0.19 ^a^	*p* = 0.255

Different superscript letters within a row indicate statistically significant differences (Tukey HSD, *p* < 0.05). Minor components (C16:1, C17:0, C17:1, C20:0, C20:1, C22:0, C24:0; each < 0.75%) are not listed.

**Table 12:** Pearson correlation coefficients (r) among oil quality parameters, based on the full set of triplicate measurements across the six treatment groups (n = 18).

	FFA	PV	K_232_	K_268_	AnV	TPM
FFA	1					
PV	0.512 *	1				
K_232_	−0.189	−0.180	1			
K_268_	−0.313	−0.533 *	−0.405	1		
AnV	0.738 **	−0.044	−0.335	0.090	1	
TPM	0.282	0.225	−0.060	−0.045	0.200	1

* *p* < 0.05 (uncorrected); ** *p* < 0.05 after Bonferroni correction for multiple comparisons (α = 0.0033). Only the FFA–AnV correlation remained significant under the stricter, fully corrected threshold.

## Data Availability

The original contributions presented in this study are included in the article/Supplementary Material. Further inquiries can be directed to the corresponding authors.

## Supplementary Materials

The following supporting information can be downloaded at: https://www.mdpi.com/article/10.3390/foods15162788/s1, Table S1: Pearson correlation matrix of the pyrolysis experiments; Table S2: Total variance explained by PCA; Table S3: Factor loadings; Figure S1: EDS results of HS-Raw; Figure S2: EDS results of HS-500; Figure S3: EDS results of HS-700; Figure S4: EDS results of HS-900; Figure S5: EDS results of HS-700 (5–10 mm); Figure S6: EDS results of HS-700 (5 °C/min); Figure S7: EDS results of HS-700 (1 h).
