# Supplementary material for: From Agricultural Waste to Sustainable Adsorbent: Characterization of Hazelnut Shell Biochar and Its Performance in Waste Frying Oil Purification
Source: Foods. 2026 Aug 8;15(16):2788. doi: 10.3390/foods15162788 (PMC13512647; doi:10.3390/foods15162788)
Supplement: Supplementary file 1 [file foods-15-02788-s001.zip › foods-4471408-supplementary.pdf]

Supplementary Materials

From Agricultural Waste to Sustainable Adsorbent: Characterization of Hazelnut Shell Biochar and Its Performance in Waste Frying Oil Purification

Ayşenur Özuysal 1, Şelale Öncü Glaue 2,* and Tolga Akcan 2,*

1 Department of Environmental Engineering, Faculty of Engineering, Dokuz Eylül University, 35390 İzmir, Türkiye; aysenur.ozuysal@deu.edu.tr
2 Food Processing Department, Efes Vocational School, Dokuz Eylül University, 35920 İzmir, Türkiye
* Correspondence: selale.glaue@deu.edu.tr (Ş.Ö.G.); tolga.akcan@deu.edu.tr (T.A.)

Contents: Table S1, Pearson correlation matrix of the pyrolysis experiments; Table S2, variance explained by the principal components; Table S3, component loadings; and Figures S1–S7, SEM/EDS fields and corresponding EDS spectra of the raw hazelnut shell and biochars.

Table S1. Pearson correlation matrix of the pyrolysis experiments (n = 7).

Variable	T	PS	RT	HR	Yield	BET	Dp	Vp	C	O	K	Ca	O/C
T	1.000	−0.152	0.802*	0.802*	−0.959**	0.824*	−0.745	0.882**	0.828*	−0.845*	0.300	−0.016	−0.862*
PS		1.000	−0.240	−0.240	0.193	0.091	0.123	0.042	0.075	−0.029	−0.816*	−0.387	0.022
RT			1.000	0.731	−0.878**	0.808*	−0.855*	0.656	0.700	−0.734	0.579	0.182	−0.764*
HR				1.000	−0.891**	0.758*	−0.853*	0.612	0.726	−0.759*	0.502	0.271	−0.783*
Yield					1.000	−0.817*	0.895**	−0.749	−0.890**	0.914**	−0.423	−0.014	0.934**
BET						1.000	−0.755*	0.867*	0.598	−0.625	0.332	0.350	−0.649
Dp							1.000	−0.471	−0.826*	0.855*	−0.515	−0.104	0.879**
Vp								1.000	0.542	−0.555	0.124	0.159	−0.568
C									1.000	−0.998**	0.083	−0.404	−0.992**
O										1.000	−0.145	0.350	0.998**
K											1.000	0.660	−0.207
Ca												1.000	0.298
O/C													1.000

Abbreviations: T, temperature; PS, particle size; RT, residence time; HR, heating rate; Yield, biochar yield; BET, BET surface area; Dp, apparent average pore diameter calculated as $D = 4V/A$; Vp, total pore volume. * $p < 0.05$; ** $p < 0.01$ (two-tailed, unadjusted). Given the limited number of pyrolysis conditions, the correlations were interpreted exploratorily.

Table S2. Total variance explained by PCA.

Component	Eigenvalue	Variance explained (%)	Cumulative variance (%)
PC1	5.01626	83.60	83.60
PC2	0.43476	7.25	90.85
PC3	0.26430	4.41	95.26
PC4	0.18664	3.11	98.37
PC5	0.09516	1.59	99.95
PC6	0.00288	0.05	100.00

The first two principal components were retained for two-dimensional exploratory visualization and together explained 90.85% of the total variance.

Table S3. Factor loadings.

Parameter	PC1 (83.60%)	PC2 (7.25%)
Temperature (°C)	**0.953**	−0.052
Residence time (min)	**0.897**	0.201
Heating rate (°C/min)	**0.896**	−0.001
Biochar yield (wt.%)	**−0.992**	0.095
BET surface area (m^2/g)	**0.876**	**0.413**
C (at.%)	**0.865**	**−0.460**

Note: Loadings with absolute values ≥ 0.40 are shown in bold.

Supplementary Figures

The EDS results are localized, semi-quantitative measurements obtained from one representative area per sample. In each figure, the left panel shows the analyzed SEM/EDS field and the right panel shows the corresponding EDS spectrum.

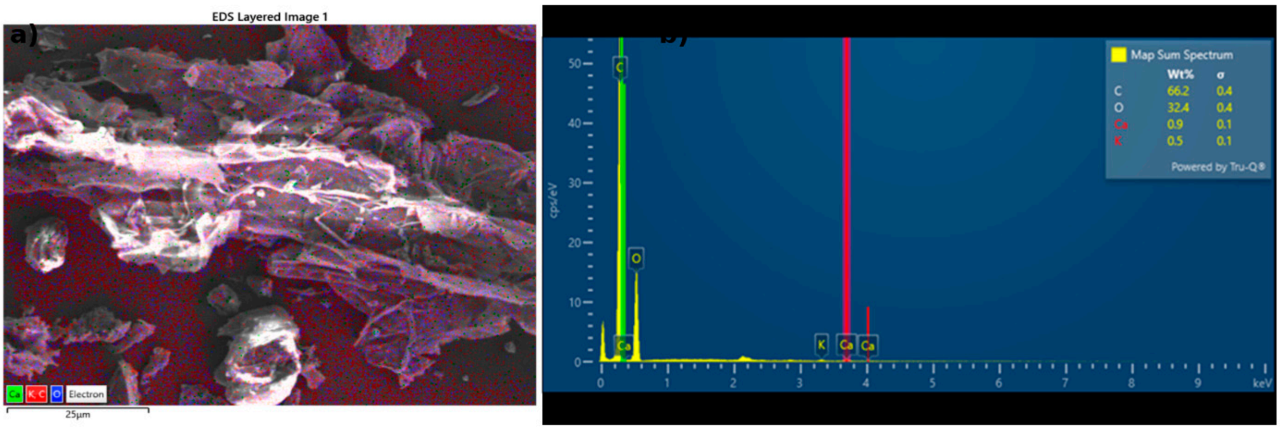

Figure S1. EDS results of HS-Raw.

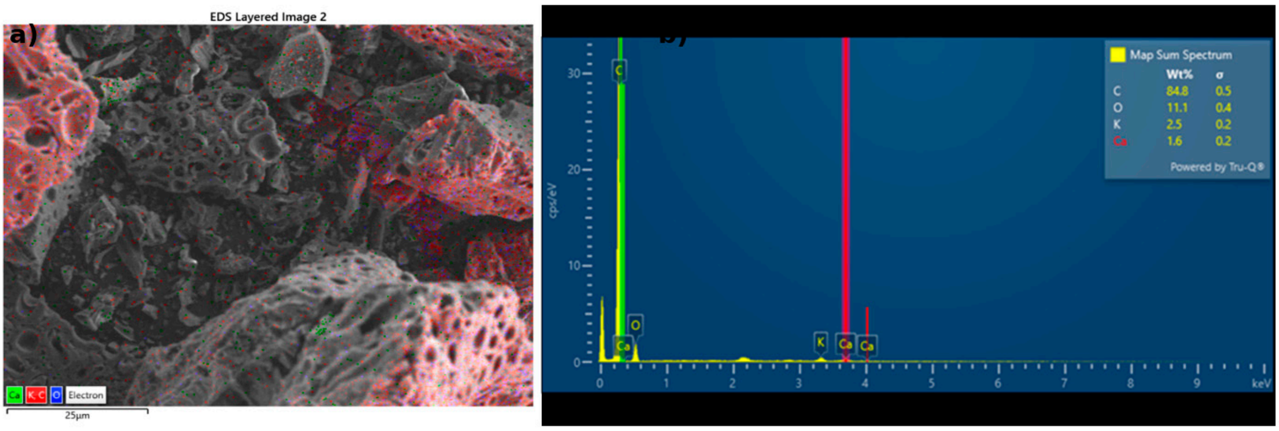

Figure S2. EDS results of HS-500.

a)

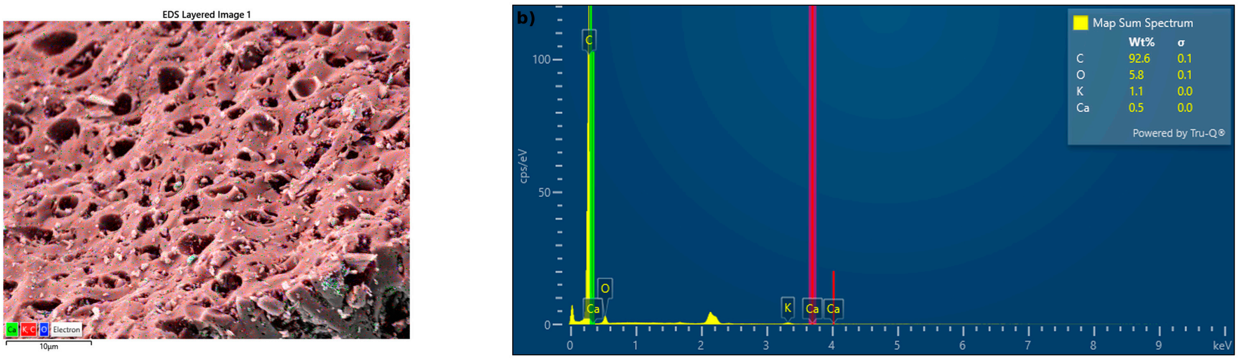

Figure S3. EDS results of HS-700.

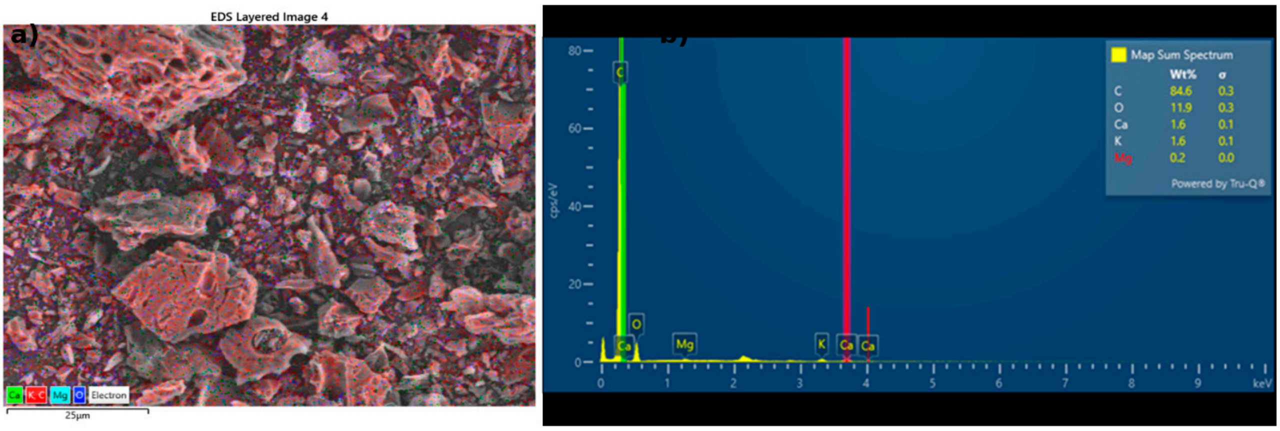

Figure S4. EDS results of HS-900.

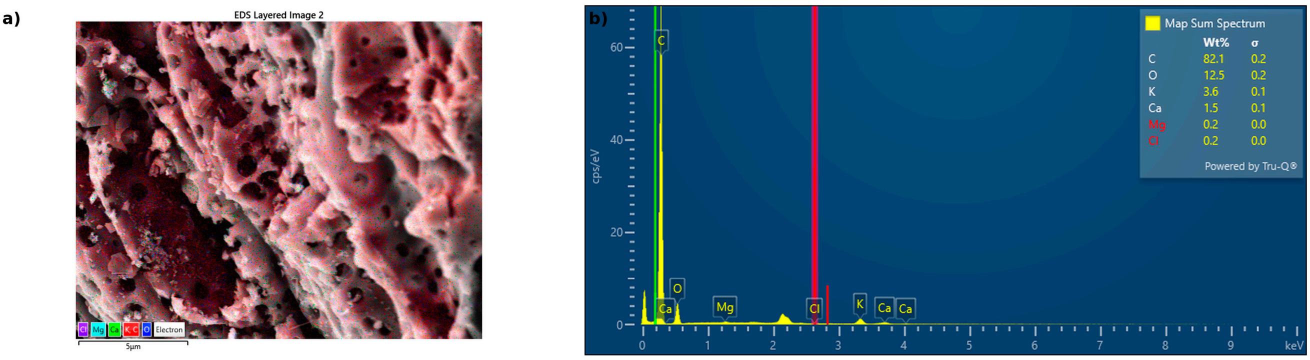

Figure S5. EDS results of HS-700 (5–10 mm).

a)

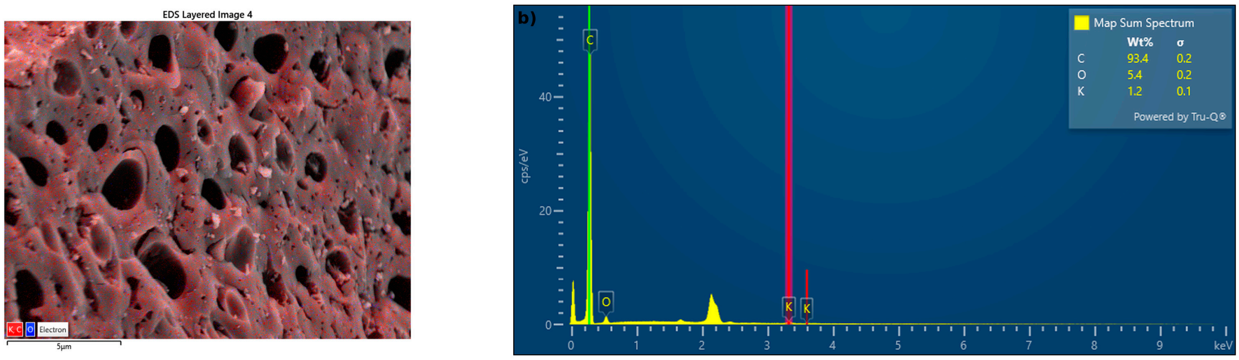

Figure S6. EDS results of HS-700 (5 °C/min).

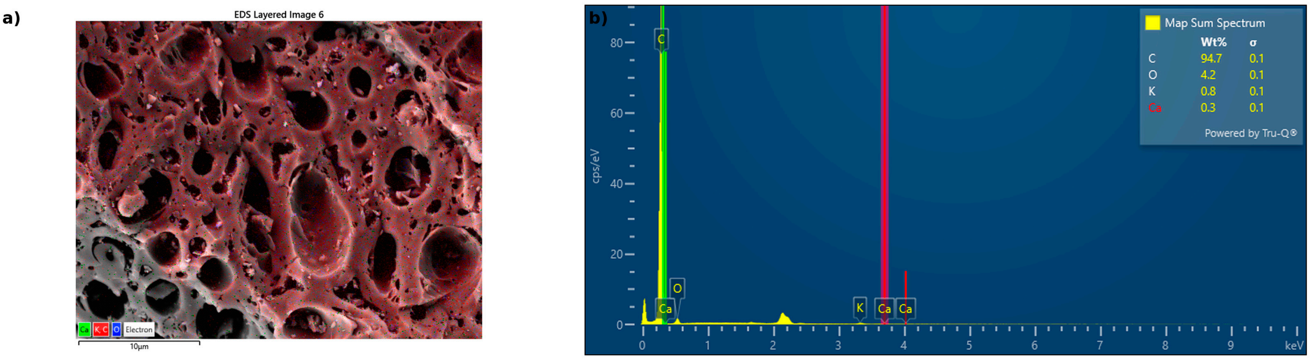

Figure S7. EDS results of HS-700 (1 h).
